# Genomewide Transcriptional Responses of Iron-Starved *Chlamydia trachomatis* Reveal Prioritization of Metabolic Precursor Synthesis over Protein Translation

**DOI:** 10.1128/mSystems.00184-17

**Published:** 2018-02-13

**Authors:** Amanda J. Brinkworth, Mark R. Wildung, Rey A. Carabeo

**Affiliations:** a School of Molecular Biosciences, College of Veterinary Medicine, Washington State University, Pullman, Washington, USA; b Laboratory of Biotechnology and Bioanalysis, College of Veterinary Medicine, Washington State University, Pullman, Washington, USA; University of California, Berkeley

**Keywords:** *Chlamydia*, microbiology, global regulatory networks, intracellular bacteria, iron reduction, stress response, stringent response, systems, transcriptional regulation, translational control

## Abstract

Iron is essential for growth and development of *Chlamydia*. Its long-term starvation in cultured mammalian cells leads to production of aberrant noninfectious chlamydial forms, also known as persistence. Immediate transcriptional responses to iron limitation have not been characterized, leaving a knowledge gap of how *Chlamydia* regulates its response to changes in iron availability. We used the fast-chelating agent 2,2′-bipyridyl (BPDL) to homogeneously starve *Chlamydia trachomatis* serovar L2 of iron, starting at 6 or 12 h postinfection. Immediate transcriptional responses were monitored after only 3 or 6 h of BPDL treatment, well before formation of aberrant *Chlamydia*. The first genomewide transcriptional response of *C. trachomatis* to iron starvation was subsequently determined utilizing RNA sequencing. Only 7% and 8% of the genome were differentially expressed in response to iron starvation at the early and middle stages of development, respectively. Biological pathway analysis revealed an overarching theme. Synthesis of macromolecular precursors (deoxynucleotides, amino acids, charged tRNAs, and acetyl coenzyme A [acetyl-CoA]) was upregulated, while energy-expensive processes (ABC transport and translation) were downregulated. A large fraction of differentially downregulated genes are involved in translation, including those encoding ribosome assembly and initiation and termination factors, which could be analogous to the translation downregulation triggered by stress in other prokaryotes during stringent responses. Additionally, transcriptional upregulation of DNA repair, oxidative stress, and tryptophan salvage genes reveals a possible coordination of responses to multiple antimicrobial and immunological insults. These responses of replicative-phase *Chlamydia* to iron starvation indicate a prioritization of survival over replication, enabling the pathogen to “stock the pantry” with ingredients needed for rapid growth once optimal iron levels are restored.

**IMPORTANCE** By utilizing an experimental approach that monitors the immediate global response of *Chlamydia trachomatis* to iron starvation, clues to long-standing issues in *Chlamydia* biology are revealed, including how *Chlamydia* adapts to this stress. We determined that this pathogen initiates a transcriptional program that prioritizes replenishment of nutrient stores over replication, possibly in preparation for rapid growth once optimal iron levels are restored. Transcription of genes for biosynthesis of metabolic precursors was generally upregulated, while those involved in multiple steps of translation were downregulated. We also observed an increase in transcription of genes involved in DNA repair and neutralizing oxidative stress, indicating that *Chlamydia* employs an “all-or-nothing” strategy. Its small genome limits its ability to tailor a specific response to a particular stress. Therefore, the “all-or-nothing” strategy may be the most efficient way of surviving within the host, where the pathogen likely encounters multiple simultaneous immunological and nutritional insults.

## Abstract (precis)

By utilizing an experimental approach that monitors the immediate global response of *Chlamydia trachomatis* to iron starvation, clues to long-standing issues in *Chlamydia* biology are revealed, including how *Chlamydia* adapts to this stress. We determined that this pathogen initiates a transcriptional program that prioritizes replenishment of nutrient stores over replication, possibly in preparation for rapid growth once optimal iron levels are restored. Transcription of genes for biosynthesis of metabolic precursors was generally upregulated, while those involved in multiple steps of translation were downregulated. We also observed an increase in transcription of genes involved in DNA repair and neutralizing oxidative stress, indicating that *Chlamydia* employs an “all-or-nothing” strategy. Its small genome limits its ability to tailor a specific response to a particular stress. Therefore, the “all-or-nothing” strategy may be the most efficient way of surviving within the host, where the pathogen likely encounters multiple simultaneous immunological and nutritional insults.

## INTRODUCTION

The sexually transmitted bacterium *Chlamydia trachomatis* infects the mucosal epithelium of the endocervix, urethra, and anogenital tract. These infections usually resolve spontaneously, and most are asymptomatic and thus underreported. Over 1.5 million cases of *C. trachomatis* genital infections were reported in the United States in 2015 alone (1). As many as 17% of females infected with *C. trachomatis* develop long-term infections in the genital tract, which can result in serious complications such as pelvic inflammatory disease (PID), fallopian-tube scarring, and ectopic pregnancy, all of which are major risk factors for tubal factor infertility (TFI) (2). Rectal infections with lymphogranuloma venereum (LGV) serovars of *C. trachomatis* can be invasive and, if untreated, can lead to complications such as proctocolitis, inguinal adenopathy, reactive arthropathy, and colorectal ulcers (3). In some patients, infection persists even after antibiotic treatment (4, 5). The ability of *C. trachomatis* to survive over the long term in some individuals despite host immunity and antibiotic treatment is not well understood and may be associated with *Chlamydia*’s ability to become persistent (6). While aberrant chlamydial forms have been identified in cervical samples, the clinical relevance of this phenomenon is not well understood (6, 7).

Chlamydiae are obligate intracellular Gram-negative bacteria that undergo a biphasic developmental cycle that includes both nonreplicative and replicative forms (8). Infection begins when the small, metabolically quiescent chlamydial elementary body (EB) binds to mucosal epithelial cells and translocates virulence factors that induce its endocytic uptake. Within 2 h of entry, the EB differentiates into its replicative form, the reticulate body (RB). Continued secretion of effectors leads to modification of the endocytic vesicle such that it avoids fusion with the lysosome and enables capture of nutrient-rich vesicles. This unique intracellular niche, called the inclusion, continues to expand as RBs replicate. In response to unknown signals present at around 24 h postinfection (p.i.). RBs then differentiate into infectious EBs, followed by EB release 36 to 72 h postinfection (8). Under conditions of exposure to certain forms of stress in cell culture (e.g., penicillin treatment, interferon gamma [IFN-γ] treatment, iron depletion, or tryptophan [Trp] depletion), RBs do not differentiate into EBs but instead enter into a state of persistence characterized by aberrant, enlarged morphology (9–13). Persistent *Chlamydia* bacteria are resistant to both antibiotics and host immunity mechanisms and can recover from this state upon removal of stress or addition of missing nutrients (14–17).

Chlamydiae have undergone reductive evolution as they have adapted to intracellular growth in mammalian cells, discarding metabolic genes responsible for synthesizing factors that could be acquired from the host (18). The core genome of *C. trachomatis* serovar L2 includes only 889 open reading frames, making *Chlamydia* dependent on its host for lipids, nucleotides, amino acids, and metal cofactors (18). Exposure of *Chlamydia-*infected cells to immune mediators, such as IFN-γ, reduces the availability of these factors and results in reduced RB division and differentiation (12, 14). IFN-γ induces intracellular depletion of tryptophan by increasing levels of indolamine 2,3-dioxygenase (IDO), which is responsible for catabolizing tryptophan into kynurenines, which cannot be utilized in tryptophan metabolism (19).

Induction of inflammatory cytokines such as IFN-γ and interleukin-6 (IL-6) in response to chlamydial infection likely causes sequestration of free iron by the activity of the mononuclear phagocytic system, which includes both cellular and systemic regulatory pathways (20–27). Readers are referred to two comprehensive reviews of the coordinated regulation of iron homeostasis by systemic and cellular mechanisms (26, 28). In the context of *Chlamydia* infection of the genital epithelium, iron availability in infected cells is likely limited by downregulation of transferrin receptor and upregulation of the iron-storage factor ferritin (29). Iron levels in the female genital tract can also fluctuate throughout the menstrual cycle, in part due to hormone-induced expression of lactoferrin (30, 31). Iron is essential for growth and development of *Chlamydia*, and its acquisition and accumulation must be carefully regulated. In mammals, readily usable ferrous iron (Fe^2+^) is tied up in molecular complexes, limiting their interaction with hydrogen peroxide to form damaging hydroxyl radicals through the Fenton reaction (32). Eukaryotic stores of ferric iron (Fe^3+^) are strictly regulated to restrict access by pathogenic bacteria (33). Extracellular bacteria such as *Pseudomonas* and *Yersinia* utilize multiple redundant iron-binding molecules called siderophores that compete with mammalian transferrin for ferrous iron (34, 35). Intracellular bacteria, such as *Mycobacterium*, *Francisella*, and *Chlamydia*, can obtain iron by subverting host vesicles that contain holo-transferrin bound to transferrin receptor (36–39). Using a combination of endocytic markers and chemical inhibitors, members of our laboratory discovered that *Chlamydia* specifically recruits transferrin-containing vesicles from the slow-recycling endocytic pathway (38). Once delivered into the inclusion, iron is likely imported into bacteria through an ABC transporter system, encoded by *ytgABCD*, which is the only known iron acquisition system in *Chlamydia* species (40–42). The C terminus of the YtgC permease, referred to as YtgR, is homologous to the *Corynebacterium* repressor DtxR and has been recently characterized as an iron-dependent repressor of the *ytgABCD* iron acquisition operon (42). A recent review highlights the differences between the iron acquisition strategies of *Chlamydia* and those of other intracellular bacteria (27).

Conversion to the aberrant phenotype in response to iron starvation reduces the infectious potential of *Chlamydia*, since only a portion of RBs recover from stress and complete development into infectious EBs once iron is added back into the media (43). Previous studies have characterized aberrant *C. trachomatis* after long-term treatment with the iron chelator deferoxamine and have detected increased expression of the iron-binding protein YtgA, indicating its role in iron uptake (41, 44, 45). However, the researchers who performed those studies added deferoxamine at the time of infection and did not monitor transcriptional or proteomic patterns until ≥24 h postinfection, making it difficult to determine whether the upregulation was part of the initial response to iron starvation. Immediate genomewide transcriptional responses to iron limitation have not yet been characterized in detail, leaving a gap in the current knowledge of how *Chlamydia* regulates its response to changes in iron availability. We utilized the chelator 2,2-bipyridyl (BPDL), which can quickly and efficiently chelate free iron from both bacterial and mammalian cells, to induce an immediate transcriptional response to iron starvation by *C. trachomatis* (43, 46–48).

This report provides the first global profile of the *C. trachomatis* transcriptional response to iron starvation. Our short-term, effective treatment regimen, in combination with deep RNA sequencing (RNA-seq), revealed the immediate response of *Chlamydia* to iron starvation in the logarithmic phase of growth when the bacteria are in the RB form. Here, we utilized this data set to map the specific biological pathways altered in response to iron starvation. Taken together, our results provide important clues to how *Chlamydia* survives iron limitation. Accumulation of metabolite precursors is prioritized over macromolecular biosynthesis. In addition, the transcriptional induction of genes involved in adaptation to other stress factors, e.g., oxidative stress and amino acid starvation, points to the inability of *Chlamydia* to tailor its transcriptional response to a specific stress. Lastly, the global transcriptomic profile of iron-starved *Chlamydia* provides valuable insights into how the biphasic developmental cycle might irreversibly switch to persistence.

## RESULTS

### Treatment optimization to detect the immediate chlamydial response to iron starvation.

**FIG 1:**
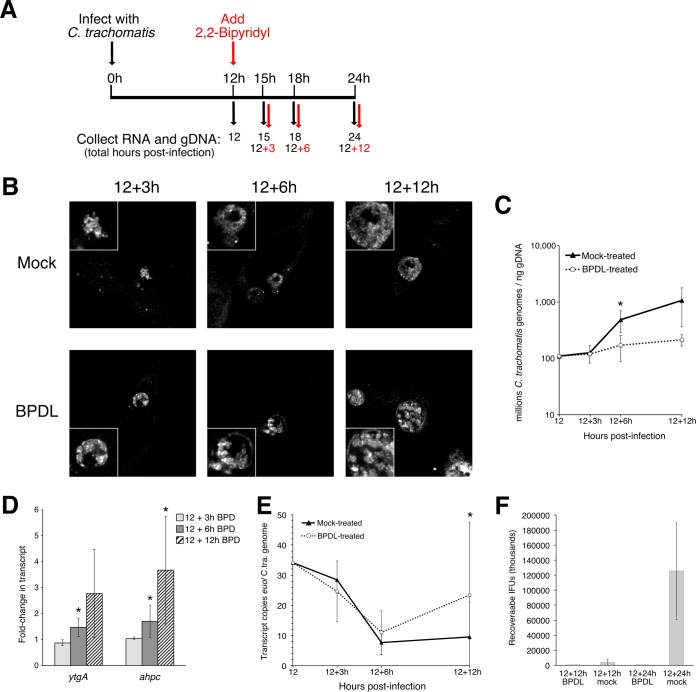
Optimization of 2,2-bipyrdyl (BPDL) treatment to induce iron responsiveness in the absence of persistence. (A) Timeline of BPDL treatment. Starting at 12 h p.i., BPDL was supplemented to culture media for 3, 6, or 12 h. (B to E) Mock-treated and BPDL-treated samples were tested for changes to morphology by confocal microscopy (B); growth by qPCR (C); iron-responsive transcription (*ytgA*, *ahpC*) (D) and transcription of the developmental marker, *euo* (C.tra., *C. trachomatis*) (E), by RT-qPCR; and levels of inclusion-forming units by IFU assay (F). Significant changes with a *P* value of <0.05 in a one-tailed Student *t* test are indicated with an asterisk and were determined on the basis of 3 biological replicates for the growth curve and 4 biological replicates for RT-qPCR.

The bivalent chelator 2,2-bipyridyl (BPDL) has been shown to deplete both ferrous iron and ferric iron from *Chlamydia*-infected cells during long-term treatment, and it induces the development of aberrant forms more consistently and homogenously than the previously used ferric iron chelator, deferoxamine (43). Here, we determined the optimal duration of BPDL treatment to induce iron-responsive transcription without inducing morphological abnormalities in *Chlamydia*. We chose to begin starvation during midcycle development (12 h p.i.) instead of at the beginning of infection for two reasons: (i) to test the response of actively replicating *Chlamydia* bacteria that are able to maximally respond to stress and (ii) to ensure that the treated and mock-treated *Chlamydia* bacteria remained in the same stage of development (RB). We monitored chlamydial morphology, growth, and transcriptional responses after 3, 6, and 12 h of BPDL treatment (Fig. 1A). Indirect immunofluorescent confocal microscopy revealed similar morphologies for the mock-treated and BPDL-treated forms for up to 12 h of BPDL treatment (Fig. 1B). Interestingly, observation of BPDL-treated cultures by light microscopy revealed an obvious decrease in Brownian movement within inclusions after 6 or more hours of treatment (data not shown). This observation is consistent with our findings showing that chlamydial growth is reduced compared to that seen with mock treatment after only 6 h of BPDL treatment, as determined by quantitative PCR (qPCR) analysis of chlamydial genomes (Fig. 1C).

We monitored the transcriptional response of the known iron-responsive genes *ytgA* and *ahpC* by reverse transcriptase quantitative PCR (RT-qPCR) to validate the iron starvation protocol (40, 41, 43, 49). Elevated (1.5-fold and 1.7-fold) transcription of both iron starvation markers was detected after only 6 h of BPDL treatment compared to the mock treatment results. Maximum differences in transcription of both markers (2.8-fold and 3.7-fold) were detected after 12 h of BPDL treatment (Fig. 1D). In the same experiment, we monitored the transcriptional profile of the early-stage gene *euo*, whose transcription decreases during late stages of normal development. Multiple persistence models have demonstrated dysregulated *euo* transcription, with high levels of *euo* mRNA detected late in development under persistence-inducing conditions (15, 43, 45). After 12 h of BPDL treatment, we observed that *euo* transcript levels remained elevated relative to the mock-treated control results, indicating dysregulated transcription or a possible delay in development (Fig. 1E, top). The idea that a delay in development occurs during longer BPDL treatment is supported by the lack of recoverable inclusion-forming units (IFUs) detected after 12 or 24 h of BPDL treatment compared to mock-treated controls (Fig. 1F), indicating a possible lack of RB-to-EB differentiation. Because 6 h of BPDL treatment is sufficient to induce iron-responsive transcription without inducing the patterns of morphology and transcription associated with persistence, we chose that as the optimal duration of iron starvation for our genomewide transcriptional studies. We also included 3 h of BPDL treatment to detect the earliest possible response to iron starvation prior to BPDL-induced changes in growth.

### Global transcriptional response of *C. trachomatis* to iron starvation during midcycle development.

The primary global response of *C. trachomatis* to midcycle iron starvation was determined by RNA sequencing (RNA-seq). We utilized an Ion Proton chip for sequencing, which allowed easy and rapid scaling of time points based on observed yields of mapped reads. This approach is relevant to the study of *Chlamydia* transcription because chlamydial mRNA represents a small proportion of the total RNA at the time points analyzed, even after significant enrichment steps. For midcycle iron starvation studies, we aimed for greater than 10× coverage of 100% of the *C. trachomatis* genome, with a minimum of 3 biological replicates per sample. The sequencing reads were trimmed to exclude adaptor sequences and polyclonal reads, followed by exclusion of reads less than 30 nucleotides (nt) in length. The remaining reads were aligned to the *C. trachomatis* genome and plasmid, with 2% to 23% of trimmed reads mapping. Average read lengths ranged from 92 to 134 nucleotides, requiring an average of 108,837 mapped reads to reach our coverage goal. A summary of the read and mapping statistics for all of our samples can be found in Table S1 in the supplemental material. Alignments, comparisons, and normalization of aligned reads were done with CLC Genomics Workbench version 9.0 according to default settings. All midcycle conditions (12 h untreated, 12 + 3 h BPDL, 12 + 3 h mock, 12 h + 6 h BPDL, 12 + 6 h mock) were compared using the CLC Genomics experiment tool, normalized by quantile scaling, and analyzed for differential gene expression levels using EdgeR statistical analysis with false-discovery-rate (FDR) calculation. Because we included ribosomal rRNA, eukaryotic mRNA, and small (<100-nt) RNA depletion steps when preparing chlamydial mRNA for RNA-seq, we also excluded tRNAs, rRNAs, and genes with <10 mean reads in a sample group prior to normalization and analysis.

10.1128/mSystems.00184-17.4 TABLE S1  Summary of RNA sequencing and mapping in this study. Download TABLE S1, PDF file, 0.04 MB. Copyright © 2018 Brinkworth et al. 2018 Brinkworth et al. This content is distributed under the terms of the Creative Commons Attribution 4.0 International license.

**FIG 2:**
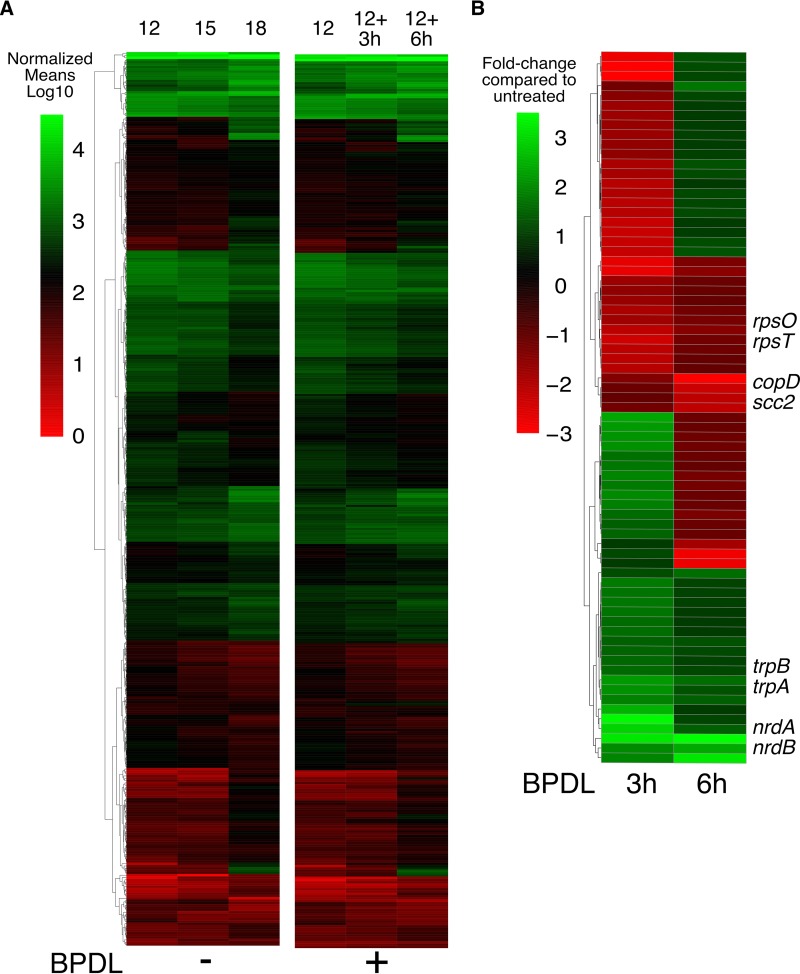
Global and differential gene expression of the midcycle response to iron starvation. The global response of *C. trachomatis* to iron starvation was detected by RNA sequencing, and reads were aligned to the genome and plasmid. (A) (Left) The untreated expression profile is displayed for all genes that changed significantly (*P* value, <0.01) during midcycle development as a heat map of log_10_-transformed normalized expression means. (Right) Levels of expression across the same genes are displayed for BPDL-treated samples. The highest and lowest expression values are displayed in green and red, respectively. (B) Genes whose expression was significantly changed in response to iron starvation, with a *P* value < 0.01, are displayed as a heat map of fold changes for BPDL-treated samples compared to mock-treated equivalent samples. The most highly upregulated and downregulated transcripts are displayed in green and red, respectively.

The genomewide profile of mock-treated and BPDL-treated gene expression during midcycle development (12 h to 18 h postinfection) is displayed as a heat map of normalized expression values (Fig. 2A). The raw and normalized data for these individual replicates can be found in Table S4. The mock treatment (left) and BPDL treatment (right) profiles were remarkably similar across all genes whose expression significantly changed during normal midcycle development of *Chlamydia* (based on comparisons of data from 12 h versus 15 h, 15 h versus 18 h, or 12 h versus 18 h; *P* value of ≤0.01). The annotated expression heat map and EdgeR comparisons for normal growth can be found in Fig. S1 and Table S2 in the supplemental material, respectively. The entire RNA-seq data set of normal development can be found in Table S3. The similarity between global expression profiles indicates that the normal development of *Chlamydia* is not dysregulated after only 3 h or 6 h of BPDL treatment. However, EdgeR analysis of BPDL-treated cultures compared to mock-treated samples (at equivalent time points postinfection) revealed that 8% (76/889) of the genome was differentially expressed after 3 h BPDL treatment and 1% (12/889) was differentially expressed after 6 h of BPDL treatment. Genes that were differentially expressed with a maximum *P* value of 0.01 are displayed in a heat map of fold change differences between BPDL-treated and mock-treated samples (Fig. 2B). Examples of decreased transcription after 3 h and 6 h of BPDL treatment include the ribosomal subunit genes *rpsO* and *rpsT* and the type III secretion genes *copB* and *scc2*, respectively. Transcription of the tryptophan salvage pathway operon *trpBA* and the ribonucleotide reductase operon *nrdAB* was significantly increased after both 3 and 6 h of treatment. Iron-responsive genes that were differentially expressed with a *P* value of <0.01 after 3 or 6 h of BPDL treatment are listed in Tables 1 and 2, respectively. The fully annotated heat map can be found in Fig. S2, and the full set of RNA-seq results for midcycle iron starvation can be found in Table S4.

10.1128/mSystems.00184-17.1 FIG S1  Annotated heat map of BPDL-treated and mock-treated gene expression in *C. trachomatis* corresponding to data in Fig. 2A. Download FIG S1, TIF file, 1.4 MB. Copyright © 2018 Brinkworth et al. 2018 Brinkworth et al. This content is distributed under the terms of the Creative Commons Attribution 4.0 International license.

10.1128/mSystems.00184-17.2 FIG S2  Annotated heat map of midcycle iron starvation corresponding to the subset in Fig. 2B. Download FIG S2, TIF file, 1.4 MB. Copyright © 2018 Brinkworth et al. 2018 Brinkworth et al. This content is distributed under the terms of the Creative Commons Attribution 4.0 International license.

10.1128/mSystems.00184-17.5 TABLE S2  Normalized means of mock-treated and BPDL-treated transcription during midcycle development of *C. trachomatis*. The mean and log_10_ mean expression values of genes that showed a significant change in gene expression during normal midcycle development (*P* value, ≤0.01) are displayed for the following EdgeR comparisons: 12 h versus 18 h, 12 h versus 15 h, and 15 h versus 18 h. These values were used to create the heat maps in Fig. 2A. Genes that had at least one value that was greater than the 4.5 threshold have an asterisk, and the values are displayed in bold. Download TABLE S2, XLSX file, 1 MB. Copyright © 2018 Brinkworth et al. 2018 Brinkworth et al. This content is distributed under the terms of the Creative Commons Attribution 4.0 International license.

10.1128/mSystems.00184-17.6 TABLE S3  Complete expression profile of *C. trachomatis* during normal development. Data corresponding to RNA sequencing reads and EdgeR analysis of normal development of *C. trachomatis* were exported from CLC Genomics Workbench 9.5.3. Samples were normalized across the entire data set by quantile scaling. rRNAs, tRNAs, and features (genes) with fewer than 10 reads in all samples were eliminated from the data set prior to normalization and EDGE analysis. Unique read data are raw values. Samples were merged from multiple RNA sequencing chips to obtain a minimum of 8× coverage. Download TABLE S3, XLSX file, 0.5 MB. Copyright © 2018 Brinkworth et al. 2018 Brinkworth et al. This content is distributed under the terms of the Creative Commons Attribution 4.0 International license.

10.1128/mSystems.00184-17.7 TABLE S4  Complete expression profile of *C. trachomatis* during midcycle iron starvation. Data corresponding to RNA sequencing reads and EdgeR analysis of iron-starved *C. trachomatis* were exported from CLC Genomics Workbench 9.5.3. Samples were normalized across the entire data set by quantile scaling. rRNAs, tRNAs, and features (genes) with fewer than 10 reads in all samples were eliminated from the data set prior to normalization and EDGE analysis. Unique read data are raw values. Samples were merged from multiple RNA sequencing chips to obtain a minimum of 8× coverage. Download TABLE S4, XLSX file, 0.6 MB. Copyright © 2018 Brinkworth et al. 2018 Brinkworth et al. This content is distributed under the terms of the Creative Commons Attribution 4.0 International license.

### Functional categorization of genes differentially expressed during midcycle response to iron starvation.

**FIG 3:**
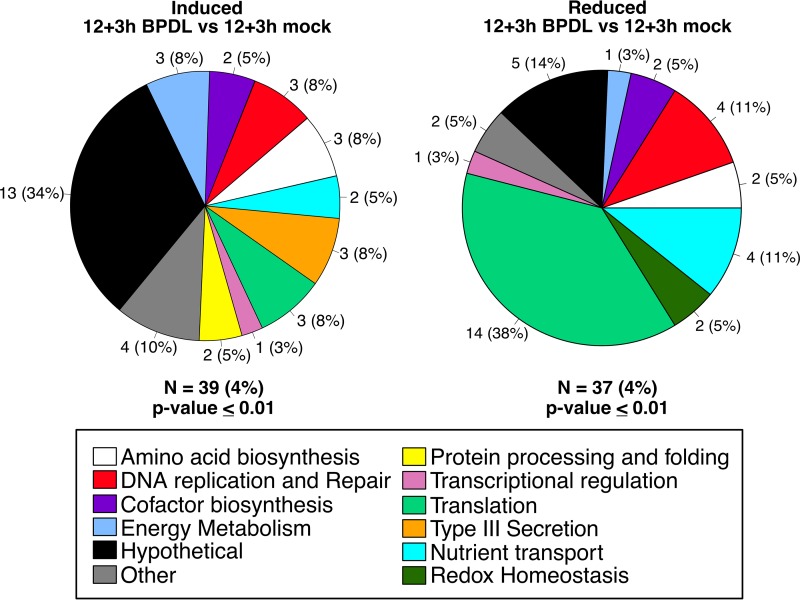
Functional categorization of midcycle response to iron starvation. Transcripts that were significantly upregulated (left) or downregulated (right) after 3 h of BPDL treatment, starting at 12 h p.i., are organized in pie charts by their functional categories. Shown adjacently to each pie slice is the number of genes in that category, and the percentages of differentially expressed genes in the category are indicated in parentheses. N = number of differentially expressed genes, and the percentage of the total genome that is represented is indicated.

Annotations and functional categories of genes differentially expressed during midcycle iron starvation were retrieved from UniProt and are listed in Tables 1 and 2. Genes differentially expressed, with a minimum *P* value of 0.01, after only 3 h of BPDL treatment are grouped by functional category of induced and reduced transcripts (Table 1; Fig. 3) (50). Of the 39 genes significantly induced after only 3 h of iron starvation, representing 4% of the genome, five categories were equally represented with 3 genes each: energy metabolism (*glgA*, *lpdA*, and *glmS*), amino acid biosynthesis (*trpA*, *trpB*, and *aroL*), DNA replication and repair (*nrdA*, *recA*, and *dnaQ*), type III secretion (*mcsC*, *CTL0085*, and *CTL0043*), and translation (*pheT*, *cysS*, and *thrS*). Of the 37 genes that were significantly reduced in response to 3 h of BPDL treatment, representing 4% of the genome, the majority (39%) are associated with translation (*prfA*, *rplW*, *rsfS*, *smpB*, *CTL0132*, *rpsK*, *rplT*, *rplN*, *CTL0680*, *rpmI*, *rmpJ*, *infa2*, *rpsT*, and *rpsO*), and 11% are associated with nutrient transport (*CTL0061*, *CTL0485*, *gltT*, and *ytgD*). Transcript levels of only 5 genes (*trpB*, *trpA*, *nrdA*, *CTL0071*, and *nrdB*) were significantly increased after 6 h of BPDL treatment, while transcript levels of 7 genes (*CTL0185*, *CTL0619*, *tsp*, and the entire *scc2-CTL0840-cobB-copD* operon) were decreased (Table 2).

**TABLE 1:** Genes differentially expressed after 3 h of BPDL treatment during midcycle development^a^

Feature ID	Locus tag	Fold change	*P* value	Annotation	Functional category	UniProtKB ID
*CTL0013*	CTL0013	3.45	3.10E−4	Hypothetical, YGGT family	Hypothetical	A0A0H3MB25
*trpB*	CTL0423	3.18	3.23E−7	Tryptophan synthase subunit B	Amino acid biosynthesis	A0A0H3MD30
*glgA*	CTL0167	2.95	1.24E−4	Glycogen synthase	Energy metabolism	B0B925
*murB*	CTL0203	2.52	6.41E−3	UDP-N-acetylenolpyruvoylglucosamine reductase	Other	B0B960
*CTL0525*	CTL0525	2.38	4.53E−5	TPR-containing domain	Hypothetical	A0A0H3MKX6
*recA*	CTL0018	2.21	1.98E−3	Recombinase A	DNA replication and repair	B0B8M5
*CTL0339*	CTL0339	2.2	2.29E−3	Phosphatidylcholine-hydrolyzing phospholipase D	Other	A0A0H3MCY1
*aroL*	CTL0621	2.13	6.56E−3	Shikimate kinase 2	Amino acid biosynthesis	A0A0H3MDG7
*hemE*	CTL0116	2.09	1.68E−3	Uroporphyrinogen decarboxylase	Cofactor biosynthesis	B0B8X3
*CTL0408*	CTL0408	2.08	1.36E−5	MIR, MAC/perforin domain-containing protein	Other	A0A0H3MGT6
*nrdA*	CTL0199	2	1.48E−5	Ribonucleoside-diphosphate reductase	DNA replication and repair	A0A0H3MCP2
*mqnD*	CTL0514	2	2.45E−3	1,4-Dihydroxy-6-naphtoate synthase	Cofactor biosynthesis	A0A0H3MC13
*CTL0704*	CTL0704	1.99	0.01	Hypothetical	Hypothetical	A0A0H3MCH5
*trpA*	CTL0424	1.94	4.15E−3	Troptophan synthase subunit A	Amino acid biosynthesis	A0A0H3MKP4
*CTL0255*	CTL0255	1.93	4.37E−3	Hypothetical	Hypothetical	A0A0H3MGJ2
*pepF*	CTL0367	1.9	6.17E−4	Endopeptidase F	Protein processing and folding	A0A0H3MKK2
*rnc*	CTL0549	1.89	0.01	RNase III	Transcriptional regulation	B0B7L3
*CTL0823*	CTL0823	1.88	7.21E−4	Hypothetical	Hypothetical	A0A0H3MLF6
*CTL0301*	CTL0301	1.81	1.32E−4	Probable cytosol aminopeptidase PepA	Protein processing and folding	B0B9F3
*CTL0884*	CTL0884	1.81	7.04E−3	Hypothetical	Hypothetical	A0A0H3MCL0
*CTL0885*	CTL0885	1.77	3.48E−3	Hypothetical effector	Type III secretion	A0A0H3MHM9
*lpdA*	CTL0820	1.76	3.80E−3	Dihydrolipoyl dehydrogenase	Energy metabolism	A0A0H3MHJ2
*CTL0096*	CTL0096	1.72	1.98E−4	Putative cation transporting ATPase	Nutrient transport	A0A0H3MCG9
*gp6D*	CTL0846	1.72	2.77E−3	Virulence plasmid pGP6-D related protein	Hypothetical	A0A0H3MLH1
*dnaQ*	CTL0513	1.69	0.01	DNA polymerase III subunit epsilon	DNA replication and repair	A0A0H3MKW6
*CTL0512*	CTL0512	1.68	4.52E−3	MCSC, secretion chaperone	Type III secretion	A0A0H3MDA7
*CTL0847*	CTL0847	1.63	6.69E−3	Hypothetical	Hypothetical	A0A0H3MCR9
*oppA3*	CTL0450	1.61	9.20E−3	Oligopeptide transporter	Nutrient transport	A0A0H3MKR3
*CTL0102*	CTL0102	1.6	7.09E−3	Putative exported protein	Hypothetical	A0A0H3MG91
*lipA*	CTL0821	1.59	8.90E−3	Lipioic acid synthase	Other	B0B8D2
*pheT*	CTL0736	1.59	0.01	Phenylalanine-tRNA ligase beta subunit	Translation	A0A0H3MHF2
*cysS*	CTL0151	1.56	2.47E−3	Cysteinyl-tRNA synthase	Translation	A0A0H3MGC2
*CTL0055*	CTL0055	1.51	7.04E−3	Hypothetical	Hypothetical	A0A0H3MAN0
*pal*	CTL0863	1.51	7.29E−3	Peptidogycan-associated lipoprotein	Other	A0A0H3MDU9
*thrS*	CTL0844	1.48	6.31E−3	Threonine-tRNA ligase	Translation	B0B8F5
*CTL0476*	CTL0476	1.48	0.01	Candidate inclusion membrane protein	Hypothetical	A0A0H3MKT3
*CTL0043*	CTL0043	0.01	0.01	Type III secretion structural protein	Type III secretion	A0A0H3MG59
*glmS*	CTL0188	1.46	0.01	Glutamine–fructose-6-phosphate aminotransferase	Energy metabolism	A0A0H3MCN0
*CTL0684*	CTL0684	1.45	8.29E−3	Hypothetical	Hypothetical	A0A0H3MCF9
						
*nusA*	CTL0352	−1.48	4.90E−3	Transcription termination factor	Transcriptional regulation	A0A0H3MGQ1
*prtA*	CTL0278	−1.56	9.49E−3	Peptide chain release factor RF1	Translation	B0B9D0
*rplW*	CTL0788	−1.58	5.43E−4	Ribosomal subunit	Translation	B0B8A0
*CTL0326*	CTL0326	−1.62	6.61E−3	YtgD, ABC transport protein, membrane permease	Nutrient transport	A0A0H3MGN6
*trpF*	CTL0581	−1.67	2.26E−3	N-(5′-Phosphoribosyl)anthranilate isomerase	Cofactor biosynthesis	B0B7P4
*folX*	CTL0878	−1.68	7.23E−3	Dihydroneopterin triphosphate 2′-epimerase	Cofactor biosynthesis	A0A0H3MCK6
*pgsA_2*	CTL0757	−1.71	1.74E−3	CDP–diacylglycerol-glycerol-3-phosphate 3-phosphatidyl-transferase	Other	A0A0H3MHG9
*sodM*	CTL0546	−1.74	1.46E−3	Superoxide dismutase	Redox homeostasis	A0A0H3MKY6
*CTL0138*	CTL0138	−1.74	4.48E−3	Ribosomal silencing factor RafS	Translation	A0A0H3MAQ8
*CTL0061*	CTL0061	−1.75	2.26E−4	Inorganic phosphate transporter	Nutrient transport	A0A0H3MG71
*CTL0486*	CTL0486	−1.77	3.48E−3	Putative membrane transport protein	Nutrient transport	A0A0H3MBY7
*gltT*	CTL0658	−1.85	3.07E−3	Sodium:dicarboxylate symport protein	Nutrient transport	A0A0H3MCC1
*smpB*	CTL0332	−1.88	0.01	SsrA-binding protein	Translation	A0A0H3MBC6
*aroA*	CTL0620	−1.91	1.85E−4	3-Phosphoshikimate 1-carboxyvinyltransferase	Energy metabolism	B0B7T5
*CTL0132*	CTL0132	−1.92	3.03E−3	UPF0109-containing putative RNA-binding protein	Translation	A0A0H3MAQ7
*CTL0548*	CTL0548	−1.98	1.14E−4	DcrA, putative nonheme Fe(II) 2-oxoglutarate	Hypothetical	A0A0H3MBY2
*secG*	CTL0606	−2.01	0.01	Protein export membrane protein SecG	Protein processing and folding	A0A0H3MH61
*rpsK*	CTL0770	−2.07	1.59E−6	Ribosomal subunit	Translation	B0B882
*rplT*	CTL0207	−2.07	1.62E−6	Ribosomal subunit	Translation	B0B964
*rplN*	CTL0780	−2.08	3.98E−3	Ribosomal subunit	Translation	B0B892
*CTL0720*	CTL0720	−2.12	5.09E−3	SWIB domain-containing protein	Hypothetical	A0A0H3MC95
*gcsH*	CTL0534	−2.14	9.08E−4	Glycine cleavage system H protein	Amino acid biosynthesis	B0B7J8
*CTL0680*	CTL0680	−2.14	1.59E−3	Putative rRNA processing peptide	Translation	A0A0H3MHB1
*rpmL*	CTL0206	−2.14	1.75E−3	Ribosomal subunit	Translation	A0A0H3MCP7
*fer*	CTL0315	−2.26	6.48E−5	Ferredoxin	Redox homeostasis	A0A0H3MCW5
*CTL0552*	CTL0552	−2.27	1.09E−3	TPR-containing domain	Hypothetical	Pseudogene
*CTL0222*	CTL0222	−2.27	6.58E−3	Hypothetical	Hypothetical	A0A0H3MK96
*infA2*	CTL0575	−2.29	6.70E−6	Translation initiation factor IF-1	Translation	A0A0H3MDD9
*rpsT*	CTL0881	−2.38	1.13E−4	Ribosomal subunit	Translation	B0B8J2
*CTL0335*	CTL0335	−2.43	0.01	Putative integral membrane protein	Hypothetical	A0A0H3MCX6
*ltuA*	CTL0631	−2.68	3.38E−3	Late transcription unit A protein	Hypothetical	A0A0H3MH76
*rpsO*	CTL0215	−2.72	4.14E−4	Ribosomal subunit	Translation	B0B972
*ndk*	CTL0762	−2.76	7.97E−4	Nucleoside diphosphate kinase	DNA replication and repair	B0B874
*pGP8-D*	pL2-02	−2.99	0.01	Virulence plasmid integrase pGP8-D	DNA replication and repair	B0BCM4
*dut*	CTL0544	−3.02	6.06E−6	Deoxyuridine 5′-triphosphate nucleotidohydrolase	DNA replication and repair	B0B7K8
*rpmJ*	CTL0154	−3.03	3.18E−4	Ribosomal subunit	Translation	B0B912
*CTL0021*	CTL0021	−3.16	0.01	Hypothetical	Hypothetical	A0A0H3MG42

^a^ FDR-corrected *P* values can be found in Table S4. Data from upregulated and downregulated genes are shown in the top and bottom halves of the table, respectively. These data were exported from CLC Genomics Workbench 9.5.3. rRNAs, tRNAs, and features (genes) with fewer than 10 reads in all samples were eliminated from the data set prior to normalization and EDGE analysis, and the data include only those genes that were differentially expressed with a significance *P* value of ≤0.01. ID, identifier.

**TABLE 2:** Genes differentially expressed after 6 h of BPDL treatment during midcycle development^a^

Feature ID	Locus tag	*P* value	Fold change	Annotation	Functional category	UniProtKB ID
*trpB*	CTL0423	2.61E−7	3.5	Tryptophan synthase subunit B	Amino acid biosynthesis	A0A0H3MD30
*trpA*	CTL0424	9.26E−6	3.21	Tryptophan synthase subunit A	Amino acid biosynthesis	A0A0H3MKP4
*nrdA*	CTL0199	3.50E−6	2.39	Ribonucleoside-diphosphate reductase	DNA replication and repair	A0A0H3MCP2
*CTL0071*	CTL0071	6.73E−3	1.73	Hypothetical	Hypothetical	A0A0H3MCG5
*nrdB*	CTL0200	9.34E−3	1.63	Ribonucleoside-diphosphate reductase	DNA replication and repair	A0A0H3MK81
						
*CTL0619*	CTL0619	1.68E−3	−1.83	Hypothetical integral membrane protein	Hypothetical	A0A0H3MH71
*copD*	CTL0842	3.17E−3	−2.13	Type III secretion system protein	Type III secretion	A0A0H3MHK7
*scc2*	CTL0839	1.37E−3	−2.18	Type III secretion system chaperone	Type III secretion	A0A0H3MLG7
*tsp*	CTL0700	1.28E−3	−2.34	Tail-specific protease	Protein processing and folding	A0A0H3MDM0
*CTL0185*	CTL0185	6.85E−3	−2.76	Hypothetical membrane protein	Hypothetical	A0A0H3MAV2
*copB*	CTL0841	6.79E−5	−2.81	Type III secretion system membrane protein	Type III secretion	A0A0H3MCF0
*CTL0840*	CTL0840	2.15E−3	−2.97	Hypothetical	Hypothetical	A0A0H3MCQ4

^a^ FDR-corrected *P* values can be found in Table S4. These data were exported from CLC Genomics Workbench 9.5.3. rRNAs, tRNAs, and features (genes) with fewer than 10 reads in all samples were eliminated from the data set prior to normalization and EDGE analysis. These data include only genes that were differentially expressed with a significance *P* value of ≤0.01.

**FIG 4:**
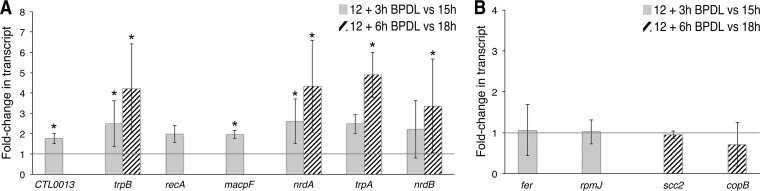
Confirmation of the midcycle response to iron starvation by RT-qPCR. Differentially expressed transcripts detected by RNA sequencing were confirmed by RT-qPCR. Data corresponding to upregulated (A) and downregulated (B) transcription are indicated as fold changes in transcripts of samples after 3 h of BPDL treatment (solid gray bars) or 6 h of BPDL treatment (striped bars) compared to mock-treated samples at equivalent time points postinfection. An asterisk indicates that the fold change was statistically significant, with a *P* value of <0.05. Statistical analysis was done with a one-tailed Student *t* test, based on results from at least 3 biological replicates.

To independently confirm the midcycle response detected by RNA sequencing, we utilized RT-qPCR. Increased transcription in response to iron starvation was confirmed for all of the transcripts tested by RT-qPCR, with the exception of *recA* (Fig. 4A). None of the tested downregulated genes were significantly reduced in expression compared to controls as determined by RT-qPCR, likely due to the fact that the genes tested were very low in abundance at the time points tested (Fig. 4B).

### Functional categorization of genes differentially expressed during early-cycle response to iron starvation.

*Chlamydia* infections of the genital tract are asynchronous. Thus, *Chlamydia* could be exposed to host-induced stress at any point in the developmental cycle. For this reason, we extended our analysis of the immediate response to iron starvation to an earlier point in the developmental cycle. *Chlamydia*-infected cells were treated with BPDL starting at 6 h postinfection, which is a time point after the initial EB-to-RB differentiation and at the beginning of the logarithmic-growth phase. RNA and genomic DNA (gDNA) were collected at 9 h postinfection for both treated and mock-treated samples. RNA-seq analyses and alignments were performed as described above. A summary of mapped reads and coverage can be found in Table S1.

**FIG 5:**
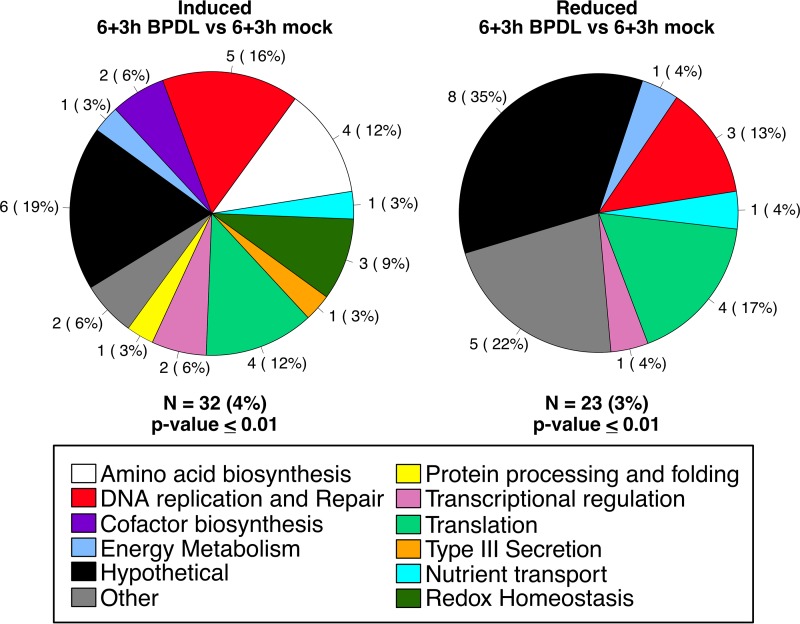
Functional categorization of the early-cycle response to iron starvation. Transcripts that were significantly upregulated (left) or downregulated (right) after 3 h of BPDL treatment, starting at 6 h p.i., are organized in pie charts by their functional categories. Adjacent to each pie slice is the number of genes in that category, and the percentages of differentially expressed genes in the category that make up the pie are indicated in parentheses. N = number of differentially expressed genes, and the percentage of the total genome that is represented is indicated.

Genes differentially expressed during the early-cycle response (6 + 3 h BPDL treatment versus 6 + 3 h mock treatment), with a maximum *P* value of 0.01, are grouped by functional categories of induced and reduced transcripts (Fig. 5). Data corresponding to the full set of differentially expressed genes and their annotations can be found in Table 3. Similarly to the results of analysis of the midcycle response, transcription of 4% of the genome, including genes involved in DNA replication and repair (*nrdA*, *nrdB*, *mutS*, *dnaQ*, and *recA*), amino acid biosynthesis (*trpB*, *trpA*, *aspC*_*1*, and *glyA*), and translation (*CTL0111*, *trpS*, *thrS*, and *aspS*), was induced during the early-cycle response to iron starvation. Uniquely, genes involved in redox homeostasis (*pdi*, *ahpC*, and *sodM*) were also upregulated in response to iron starvation starting at 6 h postinfection but not during the midcycle response. Of the 23 genes with reduced transcription during the early-cycle response to iron starvation (3% of the genome), 17% are associated with translation (*rplW*, *prfA*, *rplC*, and *ftsY*) and 13% with DNA replication and repair (*pGP8D*, *amn*, and *dnaX_1*).

**TABLE 3:** Genes differentially expressed after 3 h of BPDL treatment during early-cycle development^a^

Feature ID	Locus tag	Fold change	*P* value	Annotation	Functional category	UniProtKB ID
*CTL0149*	CTL0149	6.71	3.71E−3	Protein disulfide isomerase	Redox homeostasis	A0A0H3MAT1
*CTL0184*	CTL0184	3.4	4.44E−5	Hypothetical inclusion membrane protein	Hypothetical	A0A0H3MBD4
*trpA*	CTL0424	3.12	3.27E−4	Tryptophan synthase subunit A	Amino acid biosynthesis	A0A0H3MKP4
*CTL0388*	CTL0388	2.93	3.02E−3	Hypothetical methyltransferase	Hypothetical	A0A0H3MKL6
*CTL0111*	CTL0111	2.89	0.000877	rRNA methyltransferase TrmA	Translation	A0A0H3MAQ4
*hemN_1*	CTL0115	2.47	1.00E−2	Coproporphyrinogen-III oxidase	Cofactor biosynthesis	A0A0H3MJY5
*mip*	CTL0803	2.37	3.97E0-04	Peptidyl-prolyl *cis*-*trans*-isomerase	Protein processing and folding	A0A0H3MDR1
*trpB*	CTL0423	2.28	2.55E−3	Tryptophan synthase subunit B	Amino acid biosynthesis	A0A0H3MD30
*lpxB*	CTL0668	2.28	0.00923	Lipid-A-disaccharide synthase	Other	A0A0H3MDJ6
*nrdB*	CTL0200	2.12	1.57E−6	Ribonucleoside-diphosphate reductase subunit B	DNA replication and repair	A0A0H3MK81
*nrdA*	CTL0199	2.09	4.10E−12	Ribonucleoside-diphosphate reductase subunit A	DNA replication and repair	A0A0H3MCP2
*CTL0874*	CTL0874	2.04	2.07E−6	CADD, PABA synthase	Cofactor biosynthesis	A0A0H3MHM3
*CTL0360*	CTL0360	2.04	8.59E−3	Hypothetical	Hypothetical	A0A0H3MKJ7
*mutS*	CTL0160	1.95	6.41E−6	DNA mismatch repair protein	DNA replication and repair	B0B918
*dnaQ*	CTL0513	1.88	2.83E−3	DNA polymerase III subunit epsilon	DNA replication and repair	A0A0H3MKW6
*CTL0164*	CTL0164	1.86	1.20E−3	Hypothetical exported protein	Hypothetical	A0A0H3MBC3
*CTL0791*	CTL0791	1.82	1.78E−7	Hypothetical membrane protein	Hypothetical	A0A0H3MCL8
*trpS*	CTL0848	1.81	1.46E−3	Tryptophan-tRNA ligase	Translation	A0A0H3MCF4
*aspC_1*	CTL0005	1.77	1.92E−5	Aminotransferase	Amino acid biosynthesis	A0A0H3MG09
*eno*	CTL0850	1.75	2.51E−3	Enolase	Energy metabolism	B0B8G1
*CTL0408*	CTL0408	1.73	3.50E−4	MIR, MAC/perforin domain-containing protein	Other	A0A0H3MGT6
*recA*	CTL0018	1.72	1.49E−3	Recombinase A	DNA replication and repair	B0B8M5
*sodM*	CTL0546	1.65	7.17E−3	Superoxide dismutase	Redox homeostasis	A0A0H3MKY6
*brnQ*	CTL0817	1.62	3.60E−4	Branched-chain amino acid transporter	Nutrient transport	A0A0H3MLF2
*greA*	CTL0004	1.59	2.63E−4	Transcription elongation factor	Transcriptional regulation	A0A0H3MAD9
*CTL0102*	CTL0102	1.58	4.63E−3	Hypothetical exported protein	Hypothetical	A0A0H3MG91
*ahpC*	CTL0866	1.52	2.54E−3	Thio-specific antioxidant peroxidase	Redox homeostasis	A0A0H3MCJ5
*thrS*	CTL0844	1.52	0.00501	Threonine-tRNA ligase	Translation	B0B8F5
*aspS*	CTL0804	1.52	6.89E−3	Aspartate-tRNA ligase	Translation	B0B8B6
*rpoD*	CTL0879	1.51	1.00E−2	RNA polymerase sigma factor RpoD	Transcriptional regulation	A0A0H3MHM6
*glyA*	CTL0691	1.47	0.00242	Serine hydroxymethyltransferase	Amino acid biosynthesis	B0B804
*sctJ*	CTL0.822	1.43	7.93E−3	Type III secretion protein	Type III secretion	A0A0H3MDS0
						
*rpoC*	CTL0566	−1.28	7.87E−3	DNA-directed RNA polymerase subunit beta′	Transcriptional regulation	B0B7N0
*pGP8-D*	L2b_RS04755	−1.36	4.91E−3	Virulence plasmid integrase pGP8-D	DNA replication and repair	B0BCM4
*rplW*	CTL0788	−1.41	0.0035	Ribosomal subunit	Translation	B0B8A0
*prfA*	CTL0278	−1.44	5.80E−3	Peptide chain release factor RF1	Translation	B0B9D0
*rplC*	CTL0790	−1.52	4.30E−4	Ribosomal subunit	Translation	B0B8A2
*CTL0061*	CTL0061	−1.52	2.95E−3	Inorganic phosphate transporter PHO4	Nutrient transport	A0A0H3MG71
*CTL0659*	CTL0659	−1.57	9.75E−4	Tetraacyldisaccharide 4′-kinase LpxK	Other	A0A0H3MC42
*CTL0473*	CTL0473	−1.57	1.22E−3	Hypothetical exported protein	Hypothetical	A0A0H3MBQ9
*incD*	CTL0370	−1.59	3.33E−3	Inclusion membrane protein D	Other	B0B9M3
*plsX*	CTL0182	−1.59	1.00E−2	Phosphate acyltransferase	Other	B0B939
*CTL0613*	CTL0613	−1.6	1.34E−3	Hypothetical inner membrane protein	Hypothetical	A0A0H3MC14
*pmpA*	CTL0669	−1.63	0.00534	Probable outer membrane protein PmpA	Other	A0A0H3ML49
*CTL0548*	CTL0548	−1.66	1.01E−3	Hypothetical nonheme Fe(II) 2-oxoglutarate	Hypothetical	A0A0H3MBY2
*CTL0541*	CTL0541	−1.67	1.00E−2	Hypothetical membrane protein	Hypothetical	A0A0H3MC35
*sucB_2*	CTL0311	−1.7	7.85E−3	Dihydrolipoyllysine-residue succinyltransferase	Energy metabolism	A0A0H3ML42
*amn*	CTL0120	−1.71	4.71E−3	AMP nucleosidase	DNA replication and repair	A0A0H3MGA3
*mrsA*	CTL0547	−1.77	1.00E−2	Phosphoglucomutase	Other	A0A0H3MC40
*ftsY*	CTL0192	−1.82	4.26E−4	Signal recognition particle receptor	Translation	A0A0H3MGE5
*CTL0609*	CTL0609	−1.85	7.29E−6	Hypothetical exported protein	Hypothetical	A0A0H3MDF7
*dnaX_1*	CTL0439	−1.92	5.15E−3	DNA polymerase III subunit gamma/tau	DNA replication and repair	A0A0H3MBM8
*CTL0314*	CTL0314	−2.04	2.67E−4	Hypothetical membrane protein	Hypothetical	A0A0H3MGM7
*CTL0430*	CTL0430	−3.85	5.46E−5	Hypothetical integral membrane protein	Hypothetical	A0A0H3MBV0
*CTL0063*	CTL0063	−3.89	2.61E−3	Hypothetical	Hypothetical	A0A0H3MCG4

^a^ FDR-corrected *P* values can be found in Table S5. These data were exported from CLC Genomics Workbench 9.5.3. rRNAs, tRNA, and features (genes) with fewer than 10 reads in all samples were eliminated from the data set prior to normalization and EDGE analysis. These data include only genes that were differentially expressed with a significance *P* value of ≤0.01.

**FIG 6:**
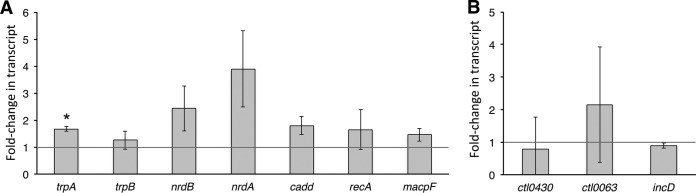
Confirmation of the early-cycle response to iron starvation by RT-qPCR. Transcripts that were significantly changed by RNA sequencing, in response to iron starvation starting at 6 h p.i., were confirmed by RT-qPCR. (A and B) Data for upregulated (A) and downregulated (B) transcription are indicated as fold changes in transcripts after 3 h of BPDL treatment in comparison to mock treatment at equivalent time points postinfection (solid gray bars). An asterisk indicates that the fold change was statistically significant, with a *P* value of <0.05. Statistical analysis was done with a one-tailed Student *t* test, based on results of two biological replicates.

Upregulation of *trpA* transcription during early-cycle iron starvation was confirmed by RT-qPCR, while only modest increases were observed for the other upregulated genes tested (Fig. 6A). Downregulation of *CTL0430*, *CTL0063*, and *incD* during iron starvation could not be confirmed by RT-qPCR (Fig. 6B). We reasoned that early-cycle responses were not detected by RT-qPCR for most of our tested genes due to the limit of detection of the technique. The raw values detected for most of our early-cycle transcripts fell at or below the lowest concentrations in our standard curves. Between 6 and 9 h postinfection, chlamydial mRNA represents a very small proportion of the total RNA. This limitation was overcome for RNA-seq experiments by depleting rRNAs and eukaryotic RNA prior to synthesizing cDNA. However, cDNA used in RT-qPCR was prepared from total RNA because mRNA enrichment would have made it impossible for us to normalize our RT-qPCR data to chlamydial genomes. The overwhelming proportion of eukaryotic RNA present in the undiluted cDNA used as the template may have impeded accurate detection of transcripts.

### Network and biological pathway analysis.

**FIG 7:**
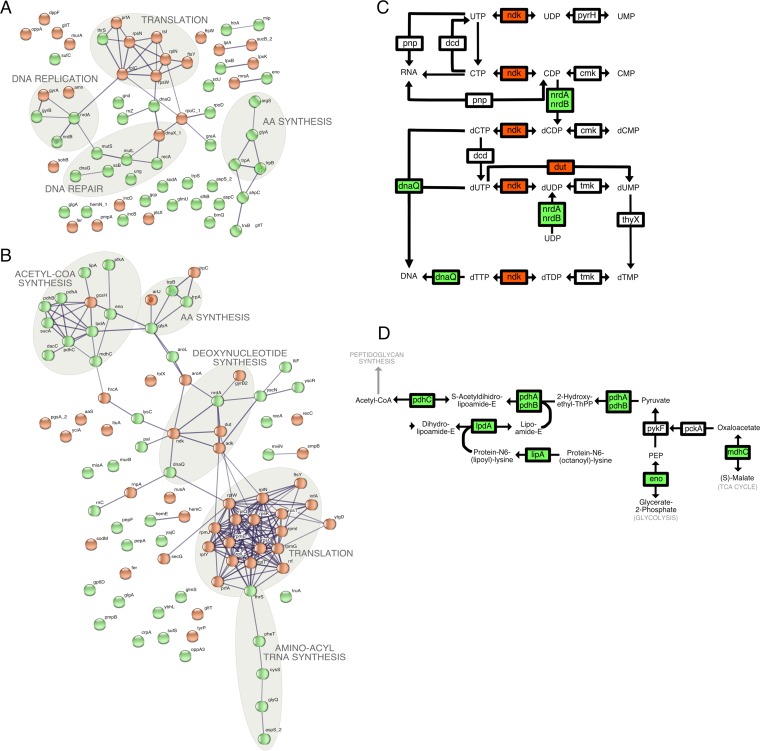
Pathway analysis of iron starvation responses. (A and B) Association networks for differentially expressed genes, with a *P* value of ≤0.05 and a minimum of 10 mapped reads, were generated for the early-cycle response (6 + 3 h BPDL) (A) and midcycle response (12 + 3 h BPDL) (B) using STRING-db v.10.5. The thickness of the lines connecting the nodes (genes) correlates with confidence of gene association, with a minimum confidence cutoff value of 0.7. (C and D) Midcycle (12 + 3 h BPDL or 12 + 6 h BPDL) clustered genes were mapped to nucleotide metabolism (C) and acetyl-CoA synthesis (D) pathways using KEGGMapper v.2.8. Data corresponding to upregulated and downregulated genes in panels C and D are shown with green and red backgrounds, respectively. Data corresponding to unchanged genes have a white background.

To further analyze the relevance of these gene expression changes to chlamydial survival, we utilized the bioinformatics tool STRING-db v.10.5 to generate networks of functionally associated genes (51). The representation of differentially expressed gene sets (*P* value, ≤0.05) corresponding to short-term iron starvation (3 h) reveals gene networks with intersecting pathway clusters (see the manually added gray circles). We chose to use the less stringent *P* value to allow entire pathways to emerge (the pathways would not have been quite as apparent with a more stringent cutoff value). Consistent with our predicted functional categories, network analysis of both early (Fig. 7A) and midcycle (Fig. 7B) responses to iron starvation revealed clusters that include amino acid biosynthesis, DNA replication and repair, and translation. Functional clustering of the midcycle response also revealed the entire cluster of genes necessary to convert pyruvate to acetyl coenzyme A (acetyl-CoA), as well as gene clusters involved in tRNA modification and charging.

The locus identifiers of genes in each identified cluster were submitted to KEGGMapper v2.8 to determine possible roles in specific biological pathways (52). For example, genes from the early-cycle DNA replication and repair cluster (Fig. 7A) were mapped to multiple pathways, including the purine metabolism (5), pyrimidine metabolism (5), mismatch repair (5), replication (4), homologous recombination (3), double-stranded break repair (2), and base excision repair (1) pathways.

### Nucleotide metabolism.

We modified the KEGGMapper output for pyrimidine metabolism to indicate the direction of change in midcycle gene expression during iron starvation (Fig. 7C). Under all iron starvation conditions, ribonucleotide reductase gene *nrdA* was upregulated. Ribonucleotide diphosphates (NDPs) bound to NrdA are converted by NrdB to deoxynucleotide diphosphates (dNDPs). These dNDPs are not likely further converted to deoxynucleotide triphosphates (dNDPs), as indicated by the downregulation of the *ndk* nucleotide diphosphate kinase gene. Available dUMP would likely be derived from the UDP pool, instead of from the dUTP pool, since transcription of the dUTP pyrophosphatase gene, *dut*, is downregulated during iron starvation. Taking the data together, these transcriptional changes would result in a net increase in levels of dNDPs, enabling rapid DNA replication when iron levels and *ndk* expression return to normal (Fig. 7C).

### Amino acid biosynthesis.

Functional clustering also indicates that *Chlamydia* prioritizes maintenance of amino acid pools during iron starvation. Multiple amino acid synthesis, interconversion, and uptake mechanisms were upregulated in response to short-term iron starvation. Transcriptional upregulation of the branched-chain amino acid transporter gene *brnQ*, the aspartate aminotransferase gene *aspC*, and the serine hydroxymethyltransferase gene *glyA* may increase the diversity of the amino acid pool such that *Chlamydia* can quickly adapt to fluctuations in amino acids. Surprisingly, the tryptophan salvage pathway genes, *trpB* and *trpA*, were consistently upregulated during short-term iron starvation. Tryptophan synthase subunit TrpB catalyzes the beta-replacement of indole with serine to form tryptophan (Trp), while TrpA facilitates the interaction of TrpB with indole (53). Their role in recovery from IFN-γ and Trp starvation stresses is well documented, but differential regulation in response to iron starvation is novel (54, 55). While the biological relevance of *trpBA* induction during iron starvation is unclear, we reason that *Chlamydia* could in fact prepare for further immune insult (e.g., IFN-γ induction of indoleamine 2,3-dioxygenase expression) by increasing intracellular Trp levels. Taking the data together, iron starvation may increase levels of serine, aspartate, glutamate, branched-chain amino acids, and tryptophan, many of which are essential for normal development (56–60). Amino acid biosynthetic genes were significantly overrepresented (4.38-fold; *P* value = 0.0464) in the set of differentially expressed midcycle genes as determined by the PANTHER overexpression test (61).

### Translation.

The largest cluster generated from STRING-db included translation factors of the midcycle response (Fig. 7B). Based on protein annotations in Uniprot and Biocyc databases, it is evident that *C. trachomatis* responds to iron starvation by shutting down factors involved in every step of translation: ribosome assembly, initiation, elongation, termination, ribosome recycling, and peptide targeting (50, 90) (Table 4). While preventing the assembly and function of translational machinery, *Chlamydia* also responds to iron starvation by increasing the levels of factors important for synthesis and modification of tRNAs, in addition to increasing transcription of *rnC*, the product of which cleaves rRNA transcripts into ribosomal subunit precursors (Table 4). Translation genes were significantly overrepresented (3.26-fold, *P* value = 0.0243) in the set of midcycle differentially expressed genes as determined by the PANTHER overexpression test (61).

**TABLE 4:** Translation factors differentially expressed during midcycle iron starvation

Gene^a^	Expression change	Locus tag	Protein ID	Annotation	Interacting component	Interaction function	Expression change (6 + 3 h BPDL)
tRNA processing							
*rnpA_1*	Decrease	CTL0153	RnP1	RNase P protein component	tRNA	Cleaves 5′ end of pre-tRNA	
*rnpA_2*	Decrease	CTL0153A	RnP2	RNase P protein component	tRNA	Cleaves 5′ end of pre-tRNA	
							
tRNA biogenesis							
*cysS*	Increase	CTL0151	CysS	Cysteine-tRNA ligase	tRNA (Cys)	Charges tRNA with cysteine	
*pheT*	Increase	CTL0736	PheT	Phenylalanine-tRNA ligase beta	tRNA (Phe)	Charges tRNA with phenylalanine	
*glyQ*	Increase	CTL0165	GlyQ	Glycine-tRNA ligase alpha subunit	tRNA (Gly)	Charges tRNA with glycine	
*aspS*	Increase	CTL0804	AspS	Aspartate-tRNA ligase	tRNA (Asp)	Charges tRNA with aspartate	Increase
*thrS*	Increase	CTL0844	ThrS	Threonine-tRNA ligase	tRNA (Thr)	Charges tRNA with threonine	Increase
*truA*	Increase	CTL0723	TruA	tRNA pseudouridine synthase A	tRNA-anticodon loop	Converts uridines at 38, 39, and 40 to pseudouridine	
*miaA*	Increase	CTL0135	MiaA	tRNA dimethylallyltransferase	tRNA-anticodon loop	Converts adenine (37) to N6- (dimethylallyl)adenosine	
							
rRNA processing							
*rnc*	Increase	CTL0549	Rnc	RNase III	30S transcript	Cleaves 30S precursor transcript to 16S and 23S	Increase
							
Subunit assembly							
*rpsO*	Decrease	CTL0215	S15	Ribosomal protein	16S rRNA	Assembly of 30S subunit	
*rpsT*	Decrease	CTL0881	S20	Ribosomal protein	16S rRNA	Assembly of 30S subunit	
*rpsK*	Decrease	CTL0770	S11	Ribosomal protein	30S subunit	Forms Shine-Dalgarno cleft	
*rplT*	Decrease	CTL0207	L20	Ribosomal protein	23S rRNA	Assembly of 50S subunit	
*rplN*	Decrease	CTL0780	L14	Ribosomal protein	23S rRNA	Forms bridge between 30S and 50S	
*rplW*	Decrease	CTL0788	L23	Ribosomal protein	23S rRNA	Forms docking site for trigger factor	Decrease
							
Initiation							
*infA2*	Decrease	CTL0575	IF-1	Initiation factor	30S-RpsA	Recruited to 30S by RpsA	
	Decrease		IF-1	Initiation factor	IF-3	IF-1 and IF-3 recruit IF-2 to 30S, IF-2 recruits mRNA and tRNA	
*rplN*	Decrease	CTL0780	L14	Ribosomal protein	23S rRNA	Forms bridge between 30S and 50S	Decrease
*rsfS*	Decrease	CTL0138	RsfS	Ribosomal silencing factor	RplN	Inhibits 70S assembly	
*rplL*	Decrease	CTL0568	L7/L12	Ribosomal protein	GTPases	Binds GTPases required for IF-3 recruitment	
							
Elongation							
*rplL*	Decrease	CTL0568	L7/L12	Ribosomal protein	GTPases	Binds GTPases required for EF-Tu and EF-G recruitment	
							
Termination							
*prfA*	Decrease	CTL0278	RF-1	Ribosome release factor		Increases termination at UAA and UAG stop codons	Decrease
*rplL*	Decrease	CTL0568	L7/L12	Ribosomal protein	GTPases	GTPase activity required for RF-3 recruitment	
							
Recycling							
*rrf*	Decrease	CTL0046	RrF	Ribosome recycling factor		Causes disassembly of stalled ribosomes	
*CTL0791*	Increase	CTL0634	HflX	GTPase HflX	50S subunit	Binds to E-site of 70S, disassembles ribosome	
							
Nascent peptide folding/ targeting							
*ftsY*	Decrease	CTL0192	FtsY	Signal recognition particle receptor	SRP-RNC	Targets nascent membrane proteins to Sec translocase	
*secG*	Decrease	CTL0606	SecG	Protein export membrane protein	SecY	Forms SecYEG translocation channel	
*smpB*	Decrease	CTL0332	SmpB	SsrA-binding protein	tmRNA	Guides tmRNA into tRNA A site, rescuing stalled ribosomes and tagging nascent peptides for degradation	
							
Unknown function							
*rpmE*	Decrease	CTL0277	L31	Ribosomal protein	23S rRNA	Unknown	
*rplQ*	Decrease	CTL0768	L15	Ribosomal protein	23S rRNA	Unknown	
*rplY*	Decrease	CTL0168	L25	Ribosomal protein	5S rRNA	Binds to 5S in central protuberance	

^a^ These genes were shown to be differentially regulated by EdgeR analysis in CLC Genomics Workbench with a *P* value of ≤0.05 during midcycle iron starvation (12 + 3 BPDL versus 15 h). Annotations and functions were retrieved from the UniProt and BioCyc databases.

### Acetyl-CoA synthesis.

Transcription of the entire set of genes necessary for conversion of pyruvate to acetyl-CoA was induced during the midcycle response to BPDL treatment (Fig. 7D). This set includes the lipoylation enzyme genes *lipA* and *lpdA* and the genes corresponding to the entire pyruvate dehydrogenase complex, *pdhABC*. In addition, transcription of the tricarboxylic acid (TCA) cycle gene *mdhC* and the glycolysis gene *eno* was induced, likely driving formation of pyruvate from different carbon sources. Acetyl-CoA can be converted to malonyl-CoA for fatty acid biosynthesis or utilized in the formation of N-acetylglucosamine-1-phosphate for peptidoglycan synthesis, both of which are required for rapid growth of *Chlamydia* (62, 63). Since the levels of transcription of the peptidoglycan-modifying enzymes encoded by *glmS* and *murB* were also increased during iron starvation, acetyl-CoA is likely used to form new peptidoglycan. Expression of fatty acid synthesis genes was unchanged during iron starvation.

Pathway analysis of both early and midcycle responses to iron starvation (Fig. 7A and B) revealed that downregulation of translation and upregulation of amino acid synthesis and nucleotide synthesis may be important for surviving this stress. Similarly, a core set of 13 genes (*trpB*, *trpA*, *nrdA*, *recA*, *dnaQ*, *CTL0704*, *CTL0102*, *thrS*, *prfA*, *rplW*, *CTL0061*, *CTL0548*, and *pGP8-D*) showed differential expression after 3 h of BPDL treatment, during both the early and midcycle responses, while *trpB*, *trpA*, and *nrdA* were upregulated in all BPDL treatments (Fig. S3). This overlap in differential gene expression data is displayed as a Venn diagram in Fig. S3.

10.1128/mSystems.00184-17.3 FIG S3  Venn diagram of differential gene expression for all BPDL treatments. Data corresponding to overlap in genes that were differentially upregulated or downregulated across multiple treatments are displayed as a Venn diagram (*P* value, ≤0.01). Download FIG S3, TIF file, 0.7 MB. Copyright © 2018 Brinkworth et al. 2018 Brinkworth et al. This content is distributed under the terms of the Creative Commons Attribution 4.0 International license.

## DISCUSSION

We monitored the immediate global transcriptional response of *Chlamydia trachomatis* serovar L2 to short-term iron starvation during early and midcycle (RB-phase) development. In contrast to previous studies of iron starvation in *Chlamydia*, our short-term treatment performed with BPDL did not cause the hallmark changes in morphology and *euo* transcription associated with persistence. This approach enabled us to detect a response specific to iron starvation as *Chlamydia* tries to adapt to stress, rather than detect the transcriptome of the aberrant bacterium. By deep RNA sequencing, we were able to identify novel primary transcriptional responses, representing 7% to 8% of the genome, after only 3 h of iron starvation with BPDL. It is possible that a portion of the detected BPDL-responsive regulon was actually due to chelation of metals other than iron. Cu^2+^ is chelated at affinities similar to those seen with Fe^2+^ and Fe^3+^, while Zn^2+^ is chelated at a level of affinity 2 to 3 logs lower than that seen with iron ions (64). We suspect that Zn^2+^ was not efficiently depleted during the short-term BPDL treatments used in this study but cannot exclude the possibility that we had detected transcriptional changes that represent responses to altered availability of other metals. It is also possible that a more immediate response could be detected with even shorter-term BPDL treatments, though we expect a longer duration is required to chelate both free iron and iron bound to protein complexes in intracellular *Chlamydia*. Since only 12 genes were differentially expressed after 6 h of BPDL treatment, a longer duration of treatment may be necessary to detect the full secondary response, which may not be obvious until the effects of the primary transcriptional response are realized at the protein level. This conjecture is supported by the fact that 6 h of BPDL treatment maintains induction of the primary response operons, *trpBA* and *nrdAB*, while reducing or delaying expression of some late-cycle genes (*scc2 CTL0840 copB copD*, *tsp*). Decreased or delayed late gene expression has also been observed during long-term iron starvation (43, 49, 65, 66).

In agreement with proteomic observations of deferoxamine-treated *C. trachomatis* after 24 h and *C. pneumoniae* after 48 h postinfection, we observed upregulation of *CTL0874* (CADD gene), *ahpC*, *eno*, *and htrA* during short-term BPDL treatment (45, 67). In contrast to previous iron starvation studies, we did not detect a significant increase in *ytgA* expression in our RNA-seq analyses. We expected the *ytgABCD* iron acquisition operon to be induced immediately in response to iron starvation, since its repression by YtgR is dependent on the presence of available iron (42). Expression of the *ytgABCD* operon peaks during midcycle development, indicating that the iron-dependent repressor YtgR may be inactive or present at low levels during the early cycle and midcycle (15, 42). It is possible that we did not observe significant differences in the expression of the operon during iron starvation because it was already maximally expressed in the mock-treated controls (see Table S3 in the supplemental material). Global detection of YtgR repression by chromatin immunoprecipitation (ChIP) sequencing or targeted analysis of specific promoters will be necessary to delineate the contribution of YtgR activity to that of the detected iron-responsive regulon. Recent work to define targets of known transcription factors in *Waddlia chondophila* discovered binding sites of YtgC by ChIP sequencing (68). Interestingly, the level of the most frequent target, *hrcA*, was also increased during iron starvation in our study, indicating that it may also be a target of YtgC in *C. trachomatis*.

Transcriptional responses to iron starvation in most bacteria typically include upregulation of iron acquisition systems and virulence factors (69–71). While expression of the *ytgABCD* operon was not upregulated during short-term iron starvation, other unidentified iron uptake and iron-dependent repression mechanisms may exist and thus could be represented in our set of iron starvation-induced genes. The virulence factor CADD (*CTL0874*) gene and the MACPF (*CTL0408*) gene were induced in both the early and midcycle responses to iron starvation. CADD overexpression induces apoptosis under conditions of expression in cultured human epithelial cells but has also been demonstrated to play a role in folate biosynthesis (72, 73). MACPF contains a domain that may enable perforin activity but so far has only been shown to undergo cleavage upon infection and become inserted into bacterial membranes (74). Several type III secretion structural components (*mcsC*, *sctJ*, *sctR*, *fliF*, *cdsN* [*CTL0043*], and *cdsD* [*CTL0033*]) and effectors (*CTL0884*, *CTL0476*, *CTL0184*, and *CTL0081*) were also transcriptionally upregulated during iron starvation, which could potentially alter interactions between the host and chlamydial inclusion.

Similarly to the upregulation of the ribonucleotide reductase operon *nrdHIEF* seen during iron starvation in *Escherichia coli* and *Yersinia pestis*, the ribonucleotide *nrdA* and *nrdB* reductase genes are consistently upregulated during under short-term iron starvation (70, 75). This upregulation indicates that deoxynucleotides may be important for *Chlamydia* to survive this stress. However, since NrdB requires iron for its function, deoxynucleotide levels may not increase until iron becomes available. Instead, high levels of inactive NrdA-B complexes may actually impede replication and development by inducing stalling at replication forks, providing a possible explanation for the decreased replication observed during iron starvation (76, 77).

The immediate transcriptional response of *Chlamydia* to iron starvation is remarkably similar to the stringent responses seen in other bacteria, which enable rapid adaptation to various stresses by diverting resources from macromolecular biosynthesis, e.g., translation, and from growth to immediate survival, often resulting in a quiescent state (78, 79). This rapid transcriptional response is achieved through synthesis of the chemical alarmone (p)ppGpp, which interacts with RNA polymerase and DksA to globally modify transcriptional activity (80, 81). During amino acid starvation in bacteria, uncharged tRNAs in the A-site of ribosomes are sensed by RelA, which responds by synthesizing (p)ppGpp from ATP and GDP or GTP (82, 83). (p)ppGpp can also be synthesized and hydrolyzed by SpoT under other stress conditions. However, since *Chlamydia* lacks the RelA and SpoT homologues necessary for (p)ppGpp synthesis, it likely evolved alternative mechanisms to reduce growth and increase survival responses during stress (17, 84, 85). Iron starvation has been shown to induce a stringent response in *Bacillus subtilis* that upregulates transcription of amino acid biosynthesis genes (86).

Multiple amino acid synthesis, interconversion, and uptake mechanisms were upregulated in response to short-term iron starvation. Surprisingly, the primary response included an increase in expression of transcripts involved in tryptophan salvage, *trpB* and *trpA*, but not in expression of the tryptophan-dependent repressor *trpR* gene. TrpR-dependent regulation of the polycistronic transcript *trpRBA* has been extensively studied during tryptophan starvation and IFN-γ treatment but rarely, if ever, in the context of iron starvation (54, 87, 88). Notably, *trpB* levels, but not *trpR* levels, were also increased under conditions of estradiol-induced persistence, suggesting that a *trpR*-independent mechanism for inducing tryptophan salvage transcription may exist (89).

Pathway analysis clearly indicates that transcripts involved in all steps of translation from initiation to ribosome recycling are downregulated during iron starvation. This reduction in translation factors might lead to an eventual shutdown or modification of translation activity that could increase survival during stress. By shutting down energy-expensive protein synthesis, ATP and GTP pools can be rerouted to immediate survival responses (tRNA charging, transcription). Similarly, iron starvation reduces the transcription of several ABC transporter genes which require ATP for their function. Uncoupled RNA and protein levels in *Chlamydia* have also been observed during IFN-γ stress (17). The apparent decrease in translation during IFN-γ exposure could be exacerbated by decreases in the levels of components of the translation machinery in response to simultaneous iron starvation. However, decreased expression of translation factors during the primary response to iron starvation may not be apparent until preexisting ribosome-protein complexes are degraded or destabilized. This may explain why ≥24 h of iron starvation is required to induce the development of aberrant RBs (43). Downregulation of translation factors during iron starvation will have to be examined at the protein level to determine its contribution to adaptation to iron starvation and development of persistence.

In contrast to downregulation of translation, iron starvation increases transcription of amino-acyl synthesis genes (*cysS*, *pheT*, *glyQ*, *aspS*, *thrS*), which are responsible for charging tRNAs with amino acids. The apparent disconnect between increased levels of aminoacyl-tRNA pools and decreased translation indicates possible survival mechanisms. Charged tRNAs might be utilized in an immediate survival response to iron starvation, prior to the turnover of ribosomal subunits. Alternatively, *Chlamydia* might accumulate charged tRNAs for recovery and resumption of development when normal levels of iron and translation factors are restored.

A major theme that emerged from our gene expression analysis is that *Chlamydia* likely perceives iron starvation as a signal to prepare for further nutrient deprivation and immune insult. Transcriptional upregulations of tryptophan salvage pathway (*trpB*, *trpA*), oxidative stress (*ahpC*, *pdi*, and *sodM*), and DNA repair (*mutS*, *mutL*, *ssb*, *ung*, *recA*) genes indicate a protective response to antimicrobial insults of the inflammatory immune response (e.g., IDO activation, reactive oxygen species). As an obligate intracellular pathogen, *Chlamydia* has undergone reductive evolution with constant selective pressure from the host immune system and its multiple antichlamydial effectors. Due to its small (~1-Mbp) genome, *Chlamydia* may not have the capability to induce a specific transcriptional response to each particular stressor, and the simultaneous deployment of stress responses may have been the most parsimonious route of adaptation to immune insult. In this case, we would expect that iron-starved *Chlamydia* would be better protected from damage by antimicrobial insults than mock-treated *Chlamydia*. Immediate transcriptional responses to other stress conditions will need to be monitored to determine if this coordination of antimicrobial responses is unique to iron starvation.

This report provides the first evidence of a global iron-dependent regulon for *C. trachomatis*. By using a system approach to delineate *Chlamydia*’s transcriptional response to iron starvation, we have been able to detect biological pathways and place them in the context of chlamydial development. These findings are novel and add to previous studies of iron-dependent transcriptional and proteomic profiling in aberrant RBs, revealing transcriptional adaptive strategies prior to the development of a persistent state. Additionally, our results include a high-resolution profile of midcycle development of *C. trachomatis* serovar L2, including relevant time points for monitoring shifts in gene expression in the early, middle, and late cycles. We expect that this data set will prove useful for future studies that seek to determine the immediate transcriptional response of *Chlamydia* to other chemical and/or nutrient stresses. Our findings include previously unrecognized shifts in energy utilization and downregulation of translation that resemble a stringency-like survival response. *Chlamydia* may utilize a two-stage approach of increasing transcription of survival genes in the short term to delay development and survive during iron starvation, followed by an eventual shutdown of translation at later times of sustained stress. The latter might account for the observed irreversibility of the persistent state during long-term starvation for iron or tryptophan.

## MATERIALS AND METHODS

### Cell culture and infection.

HeLa monolayers were infected with *C. trachomatis* strain L2 434/Bu in 6-well plates at a multiplicity of infection (MOI) of 2 for RNA and genomic DNA (gDNA) collection experiments and on coverslips in 24-well plates for morphology studies. Cells were grown in Dulbecco’s modified Eagle’s medium (DMEM) supplemented with 10% fetal bovine serum (FBS), 2 mM glutamine, and 10 µg/ml gentamycin in 5% CO_2_ at 37°C. HeLa cells used in this study were started from P1 stocks from ATCC and were regularly checked for contamination by DAPI (4′,6-diamidino-2-phenylindole) staining and the use of a Universal Mycoplasma Detection kit (ATCC).

### RNA sequencing.

RNA was collected and pooled from 2 or 4 T75 flasks of *C. trachomatis*-infected HeLa monolayers that had been treated with 100 µM 2,2-bipyridyl (BPDL) starting at 6 or 12 h postinfection (6 h + 3 h BPDL, 12 h + 3 h BPDL, 12 h + 6 h BPDL) and from mock-treated samples at equivalent time points postinfection (9 h, 12 h, 15 h, 18 h). RNA was purified using a RiboPure Bacteria (Ambion) kit per the instructions of the manufacturer. Total RNA was further enriched for transcripts over 100 nucleotides in length by the use of a MegaClear kit (Ambion). Mammalian transcripts and rRNAs were removed using a MicrobEnrich kit (Ambion), and bacterial rRNAs were removed using a MicrobExpress kit (Ambion), repeating 2 to 3 times. The integrity and quantity of total and depleted RNA were monitored with an AATI fragment analyzer. cDNA libraries were prepared with Ion Total RNA-seq kit V2, sequencing beads were prepared using an Ion Chef system, and sequencing was performed on an Ion Proton chip with HiQ chemistry. Primary sequence analysis and trimming and binning of reads were performed using Torrent Suite Software version 5.0.5. Remaining reads were mapped to the combined core genome of *C. trachomatis* strain L2 434/Bu (GenBank accession no. AM884176) and the plasmid of *C. trachomatis* L2b CS784/08 (NZ_CP009926) using CLC Genomics Workbench 9, requiring reads be at least 30 nucleotides in length, with default alignment parameters.

The EdgeR algorithm in CLC Genomics was used to determine differential gene expression levels during development and iron starvation, assuming a false-discovery rate of 10% and *P* values of ≤0.05. tRNAs and ribosomal RNAs were filtered from the reads to account for differences in depletion efficiency, and only genes with at least 5 mapped reads were included in the analysis. Differentially expressed genes were confirmed for selected transcripts by RT-qPCR.

### qPCR and RT-qPCR.

*C. trachomatis*-infected HeLa monolayers were treated with 100 µM BPDL starting at 6 or 12 h hours postinfection (6 h + 3 h BPDL, 12 h + 3 h BPDL, 12 h + 6 h BPDL) and mock-treated samples at equivalent time points postinfection (6 h, 9 h, 12 h, 15 h, 18 h). RNA and gDNA were collected with RiboPure Bacteria and the DNeasy Blood and Tissue (Qiagen) kits, respectively. cDNA was generated with Superscript IV reverse transcriptase (Life Technologies, Inc.) using 200 to 500 ng RNA per the instructions of the manufacturer, except with the use of random nonamers instead of hexamers. Transcripts were amplified with a PowerUp SYBR green system from undiluted cDNA for early-cycle samples (6 to 9 h) or diluted 1:10 in 10 mM Tris for midcycle samples (12 to 18 h) and detected with an Applied Biosystems 7300 RT-qPCR system.

### Chlamydial morphology.

Chlamydiae were monitored for 3, 6, or 12 h for changes in morphology in response to mock treatment or treatment with 100 µM BPDL starting at 12 h postinfection. Infected cultures were fixed on coverslips and stained with pooled human serum (Sigma; H4522) at 1:750 followed by goat anti-human antibody conjugated to Alexa Fluor 488 (Thermo Fisher) at 1:1,000. DNA was stained with DAPI at 5 µg/ml. Images were taken on a Leica SP8 confocal microscope with a 63× oil objective and 4× zoom.

### IFU assay.

Chlamydiae were monitored starting at 12 h postinfection for 12 or 24 h for changes in infectivity in response to mock treatment or treatment with 100 µM BPDL at an MOI of 1. Infected cultures were scraped into 300 μl SPG (succinic acid, sodium dihydrogen phosphate, glycine) and stored at −80 C for later testing. Thawed lysates were serially diluted into complete DMEM, centrifuged onto HeLa monolayers in 24-well plates, washed with Hanks balanced salt solution (HBSS), and allowed to infect for 24 h. Infected cultures were fixed and stained with pooled human serum at 1:750 followed by goat anti-human antibody conjugated to Alexa Fluor 488 (Thermo Fisher) at 1:1,000. Inclusions were counted by fluorescence microscopy, and levels of inclusion-forming units (IFU) were calculated as previously described.

### Visual analysis of differentially expressed genes.

Functional categories were assigned for all genes differentially regulated with a *P* value of ≤0.01 by referring to the GO terms listed on UniProt. Pie charts were generated using the “pie” function in Rstudio. Heat maps were generated in Rstudio using the package “pheatmaps,” with parameters set to average clustering and Euclidean distance. The PANTHER overexpression test was done in PANTHER v12.0 on differentially regulated gene sets (total) with *P* values of ≤0.01, using the default parameters and Bonferroni correction. Pathway analysis was performed on differentially regulated genesets with *P* values of ≤0.05 and a minimum of 10 mapped reads, with STRING-db v.10.5 set to a confidence value ≥0.7. StringDB maps were slightly modified to make space to increase font size, to indicate the direction of change by color coding, and to add pathway labels without altering network relationships. Clustered genes detected with StringDB were further analyzed using KeggMapper v.2.8, and pathway maps were generated based on KeggMapper output using Affinity Designer v1.4.1.

### Data availability.

Raw and processed sequencing files were submitted to the NCBI Gene Expression Omnibus (GEO) as a Superseries, and the midcycle and early-cycle projects can be found using accession number GSE106763.

10.1128/mSystems.00184-17.8 TABLE S5  Complete expression profile of *C. trachomatis* during early-cycle iron starvation. Data corresponding to RNA sequencing reads and analysis of iron-starved *C. trachomatis* were exported from CLC Genomics Workbench 9.5.3. Samples were normalized across the entire data set by quantile scaling. rRNAs, tRNAs, and features (genes) with fewer than 10 reads in all samples were eliminated from the data set prior to normalization and EDGE analysis. Unique Read data are raw values. Samples were merged from multiple RNA sequencing chips to obtain a minimum of 8× coverage. Download TABLE S5, XLSX file, 0.2 MB. Copyright © 2018 Brinkworth et al. 2018 Brinkworth et al. This content is distributed under the terms of the Creative Commons Attribution 4.0 International license.

10.1128/mSystems.00184-17.9 TABLE S6  Primers used in this study. Download TABLE S6, PDF file, 0.02 MB. Copyright © 2018 Brinkworth et al. 2018 Brinkworth et al. This content is distributed under the terms of the Creative Commons Attribution 4.0 International license.
