# Supplementary material for: Genomewide Transcriptional Responses of Iron-Starved Chlamydia trachomatis Reveal Prioritization of Metabolic Precursor Synthesis over Protein Translation
Source: mSystems. 2018 Feb 13;3(1):e00184-17. doi: 10.1128/mSystems.00184-17 (PMC5811630; doi:10.1128/mSystems.00184-17)
Supplement: TABLE S1 [file sys001182180st1.pdf]

Supplemental Table 1. Summary of RNA-sequencing and mapping in this study.

Sample	Total reads	Mapped reads (%)	Average read length	Coverage	Unique reads (%)	Gene reads (%)	Intergenic reads (%)
6+3BPD_01	31,783,753	94042 (0.3)	111.7	10.0	55784 (59)	72,201 (77)	21,841 (23)
6+3BPD_02	25,001,608	102978 (0.4)	100.6	9.9	62238 (60)	80076 (78)	22902 (22)
6+3BPD_03	26,362,915	81,301 (0.3)	111.6	8.7	42734 (53)	64,148 (79)	17153 (21)
9h_01	26,391,673	76603 (0.3)	104.3	7.6	48,904 (64)	59,462 (78)	17,141 (22)
9h_02	18,899,700	126725 (0.7)	111.1	13.5	82938 (65)	101,114 (80)	25,611 (20)
9h_03	24,659,022	110,564 (0.5)	119.6	12.6	59,941 (54)	88,214 (80)	22,350 (20)
12h_01	11,406,647	1,183,621 (10)	133.7	151.0	1014565 (86)	972,501 (82)	211,120 (18)
12h_02	18,794,400	1,826,559 (10)	132	230.0	1,532,588 (84)	1,502,698 (82)	323,861 (18)
12h_03	6,105,409	674,170 (11)	92.7	59.7	599,579 (89)	541,378 (80)	132,792 (20)
12h_04	5,490,040	275,903 (5)	122.3	32.2	228,625 (83)	216,560 (78)	59,343 (22)
12h_05	5,238,854	164239 (3)	124.1	19.5	127329 (78)	130,139 (79)	34,046 (21)
12+3BPD_01	12,936,290	1,446,925 (11)	123.6	170.9	1,320,489 (91)	1,2241,509 (86)	205,416 (14)
12+3BPD_02	14,147,695	1054535 (7)	129.6	130.6	962069 (91)	902519 (86)	152,016 (14)
12+3BPD_03	8,479,156	791,034 (9)	97.8	73.9	685,947 (87)	644,706 (82)	146,328 (18)
12+3BPD_04	4,628,453	1,044,587 (23)	131.2	131.0	870,713 (83)	812,561 (78)	232,026 (22)
12+3BPD_05	4,817,344	536875 (11)	122	62.6	418,855 (78)	421,924 (79)	114,951 (21)
15h_01	29,595,055	5499663 (19)	129.8	682.2	5106298 (93)	4,947,981 (90)	551,682 (10)
15h_02	22,517,134	2,643,605 (12)	117.3	60.2	915,409 (35)	2,348,719 (89)	294886 (11)
15h_03	11,378,770	239,110 (2)	100.3	22.9	112,935 (47)	202,415 (85)	36,695 (15)
15h_04	3,758,844	115863 (3)	106.5	11.8	73,387 (63)	95,335 (82)	20,528 (18)
12+6BPD_01	12,105,447	1,723,006 (14)	117.9	194.1	1,419,066 (82)	1,421,098 (82)	301,908 (18)
12+6BPD_02	14,751,746	1,131,903 (8)	111.6	120.7	912,764 (81)	907,278 (80)	224,625 (20)
12+6BPD_03	13,949,255	2,024,141 (15)	96	185.7	1,812,201 (90)	1666346 (82)	357795 (18)
18h_01	18,794,400	1,826,559 (10)	109.2	190.6	1,532,588 (84)	1,502,698 (82)	323,861 18)
18h_02	13,002,450	553,409 (4)	117.3	62.0	470,659 (85)	454,119 (82)	99,290 (18)
18h_03	10,676,991	1,589,307 (15)	91.9	139.6	1,441,562 (31)	1,287,668 (81)	301,639 (19)
