# Supplementary figures and images for: Genomewide Transcriptional Responses of Iron-Starved Chlamydia trachomatis Reveal Prioritization of Metabolic Precursor Synthesis over Protein Translation
Source: mSystems. 2018 Feb 13;3(1):e00184-17. doi: 10.1128/mSystems.00184-17 (PMC5811630; doi:10.1128/mSystems.00184-17)

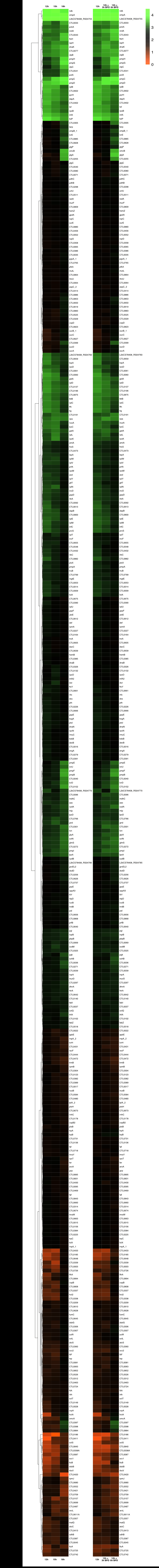

Supplement: FIG S1 [file sys001182180sf1.tif]

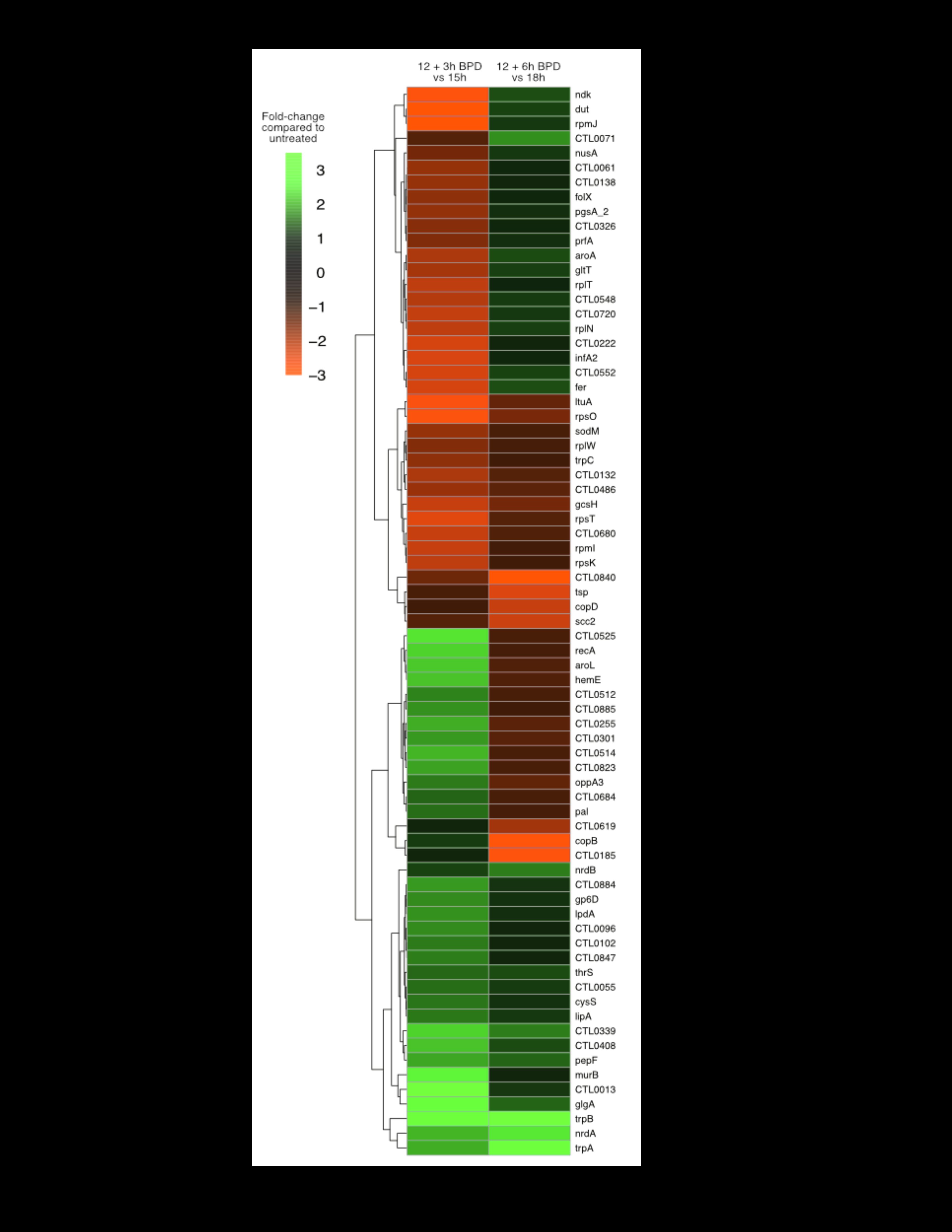

Supplement: FIG S2 [file sys001182180sf2.tif]

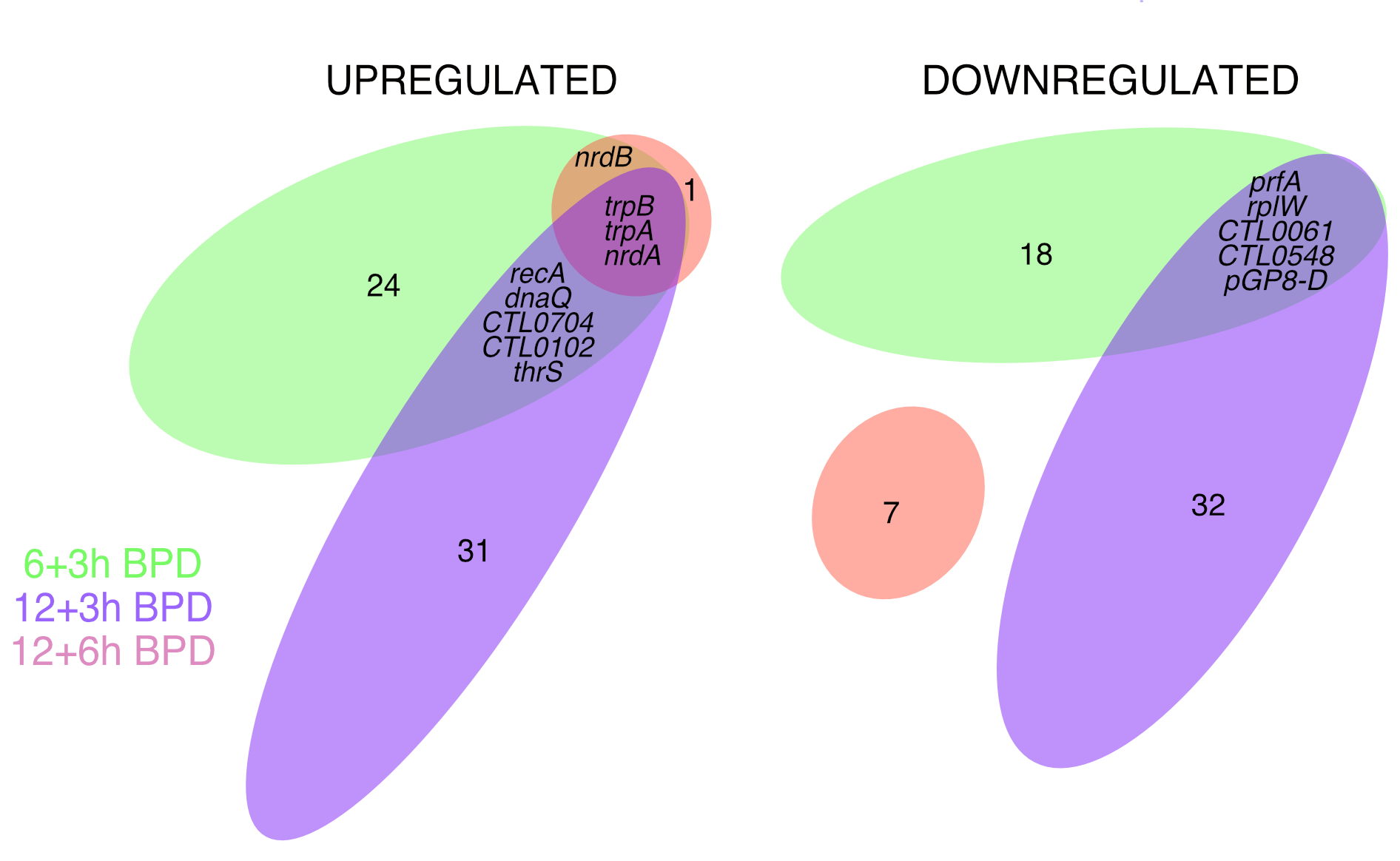

Supplement: FIG S3 [file sys001182180sf3.tif]
