# Supplementary material for: Genomewide Transcriptional Responses of Iron-Starved Chlamydia trachomatis Reveal Prioritization of Metabolic Precursor Synthesis over Protein Translation
Source: mSystems. 2018 Feb 13;3(1):e00184-17. doi: 10.1128/mSystems.00184-17 (PMC5811630; doi:10.1128/mSystems.00184-17)
Supplement: TABLE S6 [file sys001182180st6.pdf]

Supplemental Table 6. Primers used in this study.

Primer name	Primers sequence (5' to 3')
q-CTL0013 F	TCGTCGCTGGGTATCTCCCTAT
q-CTL0013 R	AAAGGGCAAAACTCCGAGACAGA
q-CTL0063_F	TACGGAGGCCATACGGTATCTT
q-CTL0063_R	ACAGGTCGAGATGGAGTAGTAGTG
q-CTL0430_F	CTACTTAACTTCCGCAGCACTCA
q-CTL0430_R	CTCTCCGCCAGTCAAAGACAA
q-CTL0704_F	AAGGGAGTACATCTTAGCCCAGAA
q-CTL0704_R	CAGGGTGGTGCTTTGGTAAAGA
q-ahpc_F	CCAGTTAGCTGGACAAACCATTC
q-ahpc_R	CGTTCCATTGACGAGGAATTGCGT
q-cadd_F	GCACATCCGCTTCTTCATGTTC
q-cadd_R	AGGAGTGGCTGCTTTGTATTCT
q-copB_F	CCTTGTGTCAAGCCAAGCATTCC
q-copB_R	GCTATCCCTTTCTGCCCTTTATG
q-euo_F	AACATAGATAGCCTGACGAGTCACA
q-euo_R	GCTGTTCCTGTTACTTCGCAAA
q-incD_F	CAGCAAGAGAGAGTTGTGGATAGG
q-incD_R	CAAGCCCGCAACAACTAGAAGA
q-fer_F	CAAGTCCCACAAACTCCTTCCATAC
q-fer_R	GGCTAAGCTCATCATTTCAGCAGAT
q-macpF_F	TGCTATGTAGATCCTCCACCTGTTC
q-macpF_R	CAGATTCCTGGTCAGCGTTTATTC
q-nrdA_F	GAGAAGAGGACGGGAGTACAGA
q-nrdA_R	CTACTTCAGACTCTTTGATGCCG
q-nrdB_F	TCAGCACCGCAGAGAGCTTG
q-nrdB_R	ATCGCAGCACGCTCGTTATAG
q-recA_F	GTGCATTGTCGTTGGATTTAGCCT
q-recA_R	GAGCATTGGCCACTATATGCGT
q-rpmJ_F	GTTAGTTCATCCATCAAAGCAGAC
q-rpmJ_R	CTTACGGTTGGGATCTTTCTTGT
q-scc2_F	ACATCCTACAGGGGTTAAGCAAC
q-scc2_R	AGACCGATAGCTTCCCGATA
q-trpA_F	AGCTCTGATTCAAGGAGGTGTTG
q-trpA_R	AGTCCCTTTGTAGAAGCGGATTG
q-trpB_F	GAGGTGGCTCCAACGCTATTG
q-trpB_R	TCCAGCGGAAATGGAGTGAG
